# *PbMYB5* transcription factor plays a role in regulating anthocyanin biosynthesis in pear (*Pyrus bretschneideri Rehd*) skin

**DOI:** 10.3389/fpls.2024.1492384

**Published:** 2025-01-14

**Authors:** Shangyun Li, Yutao Yang, Zhiwei Zhou, Xuan Zhou, Diya Lei, Ruiyuan He, Yunting Zhang, Jiliang Zhang, Yuanxiu Lin, Yan Wang, Mengyao Li, Wen He, Qing Chen, Ya Luo, Xiaorong Wang, Haoru Tang, Yong Zhang

**Affiliations:** College of Horticulture, Sichuan Agricultural University, Chengdu, China

**Keywords:** MYB transcription factor, lignin, anthocyanin, proanthocyanidins(PAs), coloration

## Abstract

The phenylacetone pathway, which encompasses flavonoids, lignin, and other compounds, is of paramount importance in determining the quality of pear fruit. Nevertheless, the precise regulatory functions of R2R3-MYB transcription factors in the metabolic pathways that regulate pear color changes remain unclear. In this study, we isolated an *R2R3-PbMYB5(PbMYB5)* transcription factor from ‘Red Zaosu’ pears and demonstrated that it influenced the expression of several genes, including *PbCAD1, PbF5H, PbLAR, PbANR*, and *PbUFGT*. The overexpression of *PbMYB5* resulted in a notable elevation in anthocyanin concentration within the pear epidermis. Further research has shown that *PbMYB5* is able to bind to *PbANS* and also has interactions with *PbbHLH3* and *PbbHLH33*.We proposed that *PbMYB5* forms a complex with *PbbHLH3, PbbHLH33*, and *PbWD40* to activate *PbANS* and promote anthocyanin accumulation. This study offers new insights into the regulation of various metabolic pathways that impact fruit coloration.

## 1 Introduction

As a global fruit, pears (*Pyrus* spp.) have great economic and food value due to their unique flavor, rich nutritional value, and diverse pigmentation (Wu et al., 2013). Anthocyanosides are important flavonoids that are not only strong antioxidants or free radical scavengers in plants that resist adversity and aid in plant reproduction but may also be important in improving pear fruit quality (Shang et al., 2011; Wen et al., 2020; Yousuf et al., 2016; Zhai et al., 2014). Anthocyanins and PAs, the main components of anthocyanidins, significantly influence the color formation of the pear skin. Moreover, lignin, a compound of the phenylpropane pathway, plays an important role in pear hardness, crispness and flavour formation. However, it is noteworthy that there is substrate competition between lignin, PAs, and anthocyanin biosynthesis. Therefore, exploring the homeostatic equilibrium of metabolite synthesis via different pathways is a priority for research on pear color formation and germplasm innovation.

Most of the biosynthesis through the phenylpropanoid pathway is well understood, and many substrates are common to the lignin, anthocyanin, and PAs biosynthetic pathways. Cinnamoyl-coenzyme A reductase (*CCR*) and chalcone synthase (*CHS*) catalyze the movement of the common substrate p-coumaroyl-coenzyme A into the lignin, PA, and anthocyanidin pathways, respectively. Anthocyanins are synthesized by anthocyanin synthase (*ANS*) and UDP-glucoflavonoid-3-glucosyltransferase (*UFGT*), and PAs are synthesized by color less anthocyanin reductase (*LAR*) and anthocyanin reductase (*ANR*) (Zhang et al., 2020). Different biosynthetic pathways are regulated by structural gene expression and the MBW ternary complex composed of R2R3-MYB, bHLH, and WD40 transcription factors (TFs), with R2R3-MYB TFs playing a central role (Jiang et al., 2023). *PyMYB10* and *PyMYB10.1* not only activate the *AtDFR* promoter but also have the ability to bind to *bHLH* cofactors such as *PybHLH*, *MrbHLH1*, *MrbHLH2*, or *AtbHLH2*. This interaction forms a complex that further boosts *AtDFR* promoter activation, ultimately leading to the positive regulation of anthocyanin biosynthesis in pear (Jiang et al., 2023). The downregulation of *PyMYB114* has been demonstrated to impede the synthesis of anthocyanin in red-skinned pears. Moreover, evidence suggests that the *ERF/AP2* transcription factor *PyERF3* interacts with *PyMYB114* and its partner *PybHLH3*, thereby regulating anthocyanin production. Furthermore, the concomitant expression of *PyMYB114* and *PyMYB10* has been observed to enhance anthocyanin synthesis. *PbMYB10b* and *PbMYB9* can regulate anthocyanins and PAs; *MYB17* directly activates the structural genes involved in anthocyanin synthesis to regulate anthocyanin accumulation (Premathilake et al., 2020; Wang et al., 2020; Wang et al., 2017). *VvMYB5a* has been shown to activate the promoters of key genes in the general flavonoid pathway, such as *VvANS*, *VvF3′5′H*, and *VvCHI*, as well as the catechin-specific gene *VvLAR1* (Deluc et al., 2006). Interestingly, it does not affect the promoter of the *VvANR* gene. When *VvMYB5b* was overexpressed in tobacco plants, it led to the increased expression of genes involved in the flavonoid pathway, resulting in higher levels of anthocyanins and PAs (Deluc et al., 2008). This demonstrates the ability of both *VvMYB5a* and *VvMYB5b* to activate the promoters of various structural genes in the grapevine flavonoid pathway (Deluc et al., 2006, 2008). *R2R3-FaMYB5* has been proven to enhance the accumulation of anthocyanin and PA by activating *F3’H* and *LAR*. Additionally, it interacts with *FaEGL3* and *FaLWD1/FaLWD1-like* to create the MYB-bHLH-WD40 complex (MBW), which enhances regulatory effectiveness (Jiang et al., 2023). The R2R3 MYB transcription factor, *MYB6*, promoted the biosynthesis of burlap anthocyanins and PAs, but inhibited burlap secondary cell wall formation (Wang et al., 2019a).

The analysis revealed that *MYB5* is involved in the synthesis of multiple pathways in different species. Simultaneously, during the *‘*Red Zaosu*’* pear fruit development process, there is a difference in peel color changes (As the fruit matures, a distinct yellow-green hue emerges on the surface of the pericarp, gradually becoming more pronounced over time.). An R2R3-MYB-mediated homeostatic regulation of lignin, PAs, and anthocyanin biosynthesis was not identified. Therefore, we proposed to investigate: (1) The color change in the pericarp of ‘Red Zaosu’ pears is a result of disruptions in the multi-pathway metabolism caused by competition between lignin, anthocyanin, and PA for substrates. (2) The transcription factor *MYB5* in the pericarp of ‘Red Zaosu’ pears plays a role in regulating the multi-pathway metabolism, and the interconnected regulation influences the color change in the pericarp. Based on this, we used the ‘Red Zaosu’ pear pericarp as the research object, *MYB5* identified as the target transcription factor, and forward and reverse genetics techniques to reveal the above problems and provide a theoretical foundation for pear color research and molecular breeding.

## 2 Materials and methods

### 2.1 Plant material

The *‘*Red Zaosu*’* and *‘*Zaosu*’* pear trees were planted in the Pear Germplasm and Innovative Resources Nursery at the Chongzhou Modern Agriculture R&D Base of Sichuan Agricultural University, located at 103°64’52’’ E and 30°55’67’’ N. The trees were of similar age, growth potential, and management level. Tobacco (Nicotiana benthamiana) was cultured under controlled environmental conditions in the Materials Room of the Plant Molecular Genetic Breeding and Biotechnology Laboratory, College of Horticulture, Sichuan Agricultural University, China, for subcellular localisation, dual luciferase experiments, and BiFC tests. The temperature was maintained at 23 ± 2°C, with a relative humidity of 80%–90% and a light-dark cycle of 16/8 h. The light intensity was 220 μmol m^-2^s^-1^. ‘Red Zaosu’ stems, leaves, buds, flowers, fruits, seeds at various developmental stages, and pear pericarp samples were collected at 25, 45, 65, 85, 105, and 115 Day after anthesis (DAF). The pear fruits were harvested, and the epidermis was removed using a scalpel. All samples were immediately frozen in liquid nitrogen and stored at –80°C. The samples were then sorted by tissue type. The *‘*Zaosu*’* and *‘*Red Zaosu*’* pears were bagged 15 d after flowering. The fruit was then collected at 30 and 90 d after bagging (DAB) for the instantaneous injection test.

### 2.2 RNA extraction and RNA sequencing

The epidermis of different regions of the fruit at 65, 85 and 105 DAF was used as the material, total RNA was extracted using the CTAB-based method (Gambino and Gribaudo, 2012) and transcribed into cDNA. The quality of the total RNA was assessed, and BioMarker company (Qingdao, China) was used to construct and sequence the libraries. Each sample included three biological replicates. The sequencing reads were analyzed using our previously described procedures (www.biocloud.net).

### 2.3 Gene cloning and molecular bioinformatics analysis

The primers listed in **Supplementary Table S1** were used to amplify and clone the genes into the pBlunt vector (Yeasen Biotechnology, Shanghai) using the mined gene sequences. The sequence information was determined by comparing and analyzing the cloned sequences in the Genome Database for Rosaceae (GDR) and National Center For Biotechnology Information databases (NCBI). The MYB structural domains were analyzed using the Pfam (pfam.xfam.org) and ESPript 3.0 online websites (Jiang et al., 2023). The ClustalW program in the MEGA 6.06 package was used for multiple sequence comparisons. A phylogenetic tree was constructed using the neighbor-joining method with 1000 bootstrap repeats.

### 2.4 Transient expression and stable transformation assays

The CDS sequence of *PbMYB5* was cloned into the expression vector pCAMBIA-35SN (35S::*PbMYB5*), and the RNA interference (RNAi) vector pRNAi-35SN (RNAi::*PbMYB5*). Transient overexpression and silencing were performed in pear fruit per the described protocol (Cong et al., 2021; Ni et al., 2023; Yao et al., 2017; Zhai et al., 2016). Each replicate included at least 10 fruits (30 and 90 DAB), with three biological replicates. The injected fruits were harvested after seven days, and the peel of the injected area was scraped using a scalpel. The harvested peel was stored at –80°C before use. For stable genetic transformation of *‘*Red zaosu*’* and *‘*Zaosu*’* healing tissues, the 35S::*PbMYB5* and RNAi::*PbMYB5* constructs were transformed into *Agrobacterium tumefaciens* GV3101. Pear healing tissues were infiltrated following the previously described method (Ni et al., 2023). Anthocyanoside synthesis was induced by culturing positive healing tissues in the dark for two weeks, followed by continuous light treatment for 96 h.

### 2.5 RT-qPCR assay

SYBR Green (TaKaRa, Dalian, China) was used to detect the PCR products on a CFX96 real-time reaction system (Bio-Rad). The housekeeping gene JN684184 (Actin) was selected and analyzed using the 2^-ΔΔCt^ method (Bai et al., 2019). The qPCR primers are listed in **Supplementary Table S2**.

### 2.6 Anthocyanin and PAs detection

The total anthocyanin content was determined using the pH difference method (Ni et al., 2023; Yao et al., 2017). PAs were detected by reaction with p-dimethylaminocinnamaldehyde (DMACA) solution (0.1% [w/v] DMACA, 90% [v/v] ethanol, and 10% [w/v] HCl) to produce a blue color, and their concentrations were determined through full-wavelength zymography using the DMACA assay, as previously described (Zhang et al., 2022). Positive healing tissues were stained for PA using DMACA solution (0.2% [w/v] DMACA, methanol: 6M HCL = 1:1 [v/v]), immersed for 48 h, rinsed with distilled water, dried and observed (Zhang et al., 2022).

### 2.7 Subcellular localization and bimolecular fluorescence complementation assays

*PbMYB5* was used to the N-terminus of eGFP in the pCAMBIA-35 S-eGFP vector (*PbMYB5*-eGFP) by homologous recombination. For BiFC, the target genes were cloned into vectors modified with pSAT1-nEYFP-C1 and pSAT1-cEYFP-N1 to produce fused YFP target proteins (Citovsky et al., 2006). The vector was transformed into *the Agrobacterium* strain GV3101. The cells were incubated overnight at 28°C in a YEP medium supplemented with the appropriate antibiotics. After centrifugation, the collected cells were resuspended in MMA solution (10 mM MES, 10 mM MgCl_2_, and 500 μM acetosyringone) and incubated at room temperature for 1 h on a shaker. The bacteria were suspended in fresh buffer and adjusted to a final density of OD 600 = 1. The suspension was injected into the dorsal axial lateral chloroplasts of the tobacco leaves using a syringe. The treated plants were kept in a greenhouse for 2–3 d and then observed under a confocal microscope (Olympus FV1000). The primers used to construct the vectors are listed in **Supplementary Table S1**.

### 2.8 Yeast two-hybrid analysis

Two target genes were cloned into the pGADT7 and pGBKT7 vectors, and positive interactions were detected using the SD/-Trp/-Leu and SD/-Trp/-Leu/-Ade/-His/+ X-α-gal/+3-AT medium. **Supplementary Table S1** lists all primers used for vector construction.

### 2.9 Yeast one-hybrid analysis

Genes were cloned into the pGADT7 vectors, target gene promoter cloning in pHIS vector, and positive interactions were detected using the SD/-Trp, and SD/-His/-Trp + 3AT, and SD/-Trp/-Leu/-His + 3-AT medium. **Supplementary Table S1** lists all primers used for vector construction.

### 2.10 Dual luciferase assay

To construct the reporter gene, promoter sequences of *CHS*, *CHI*, *F3H*, *DFR*, *LAR*, *ANR*, and *UFGT* were cloned from the ‘Red Zaosu’ pericarp and inserted into the pGreenII0800-LUC vector. 35S::*PbMYB5* was used as an effector. Empty pCAMBIA-35SN was used as a negative control. The vector was transformed into *Agrobacterium* GV3101 (pSoup-p19). Dual-luciferase transient expression assays were performed as described (Liu et al., 2008). Firefly luciferase (LUC) and Renilla luciferase (REN) were detected using the Dual-Luciferase Reporter Gene Assay Kit (Yeasen Biotechnology, Shanghai) according to the manufacturer’s instructions. **Supplementary Table S1** lists all primers used for vector construction.

### 2.11 Statistical analysis

Unless otherwise noted, all data were analyzed using the IBM SPSS Statistics 23 software. Statistically significant differences between samples were determined using Student’s t-test (P < 0.05).

## 3 Results

### 3.1 Bioinformatic identification and analysis of the R2R3 transcription factor *PbMYB5*

**Figure 1:**
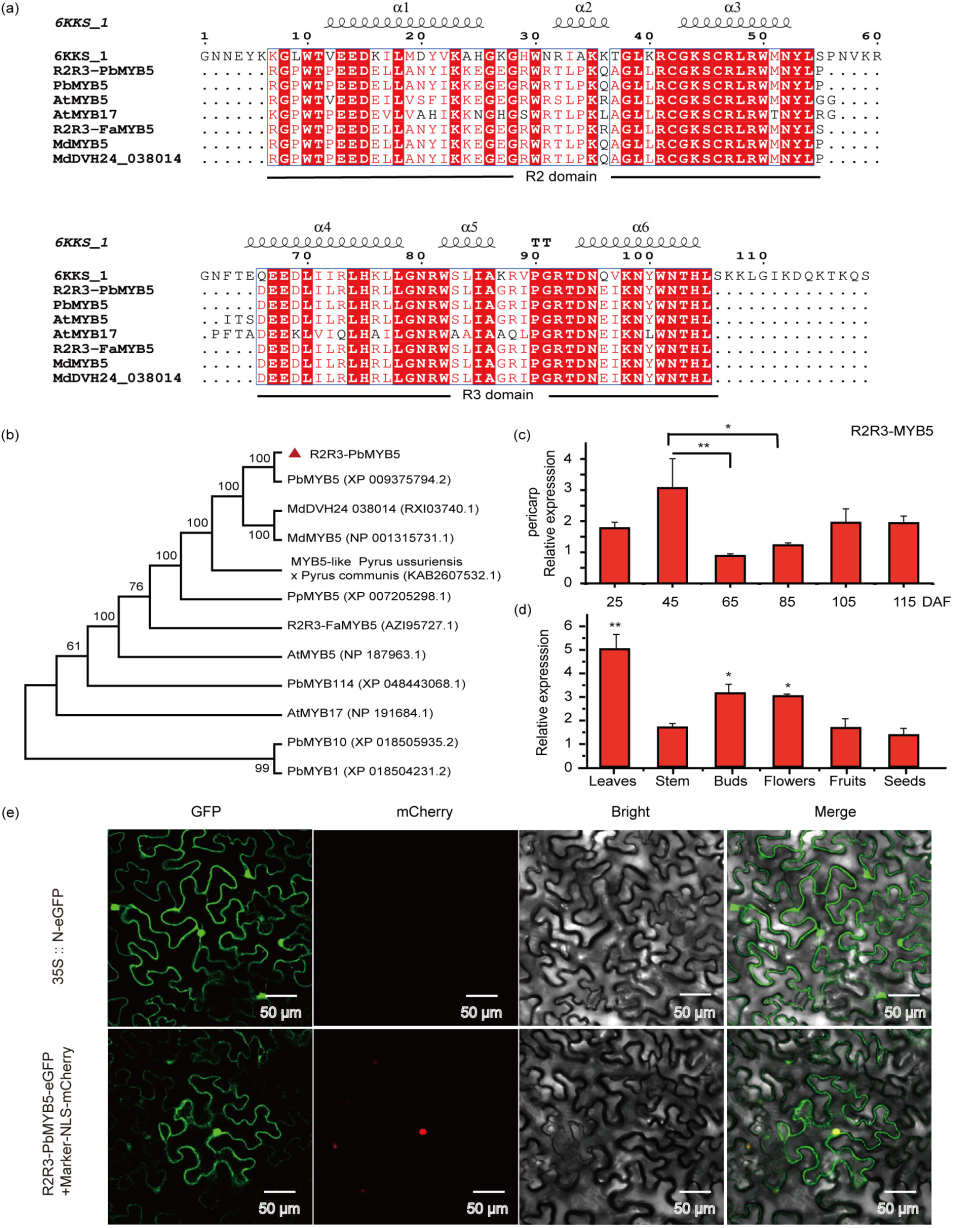
Bioinformatic analysis and subcellular localization of *PbMYB5*. **(A)** Amino acid sequence comparison and analysis of conserved structural domains. Multiple sequence alignments of *MYB5* were performed through ClustalW and the phylogenetic tree was constructed using the neighbor-joining method with 1,000 bootstrap replicates. Accession IDs (GenBank): *PbMYB5* (Pyrus x bretschneideri), XP_009375794.2; *MdDVH24_038014* (Malus domestica), RXI03740.1; *MdMYB5* (Malus domestica), NP_001315731.1; *MYB5-like* (Pyrus ussuriensis x Pyrus communis), KAB2607532.1; *PpMYB5* (Prunus persica), XP_007205298.1; *VvMYB5a* (Vitis vinifera), RVW48712.1; *VvMYB5b* (Vitis vinifera), NP_001267854.1; *R2R3-FaMYB5* (Fragaria x ananassa), AZI95727.1; *PtMYB5* (Populus tomentosa), KAG6787562.1; *PtMYB6* (Populus tomentosa), AHH34325.1; *AtMYB5* (Arabidopsis thaliana), NP_187963.1; *AtMYB17* (Arabidopsis thaliana), NP_191684.1; *PbMYB114* (Pyrus x bretschneideri), XP_048443068.1; *PbMYB10* (Pyrus x bretschneideri), XP_018505935.2; *PbMYB1* (Pyrus x bretschneideri), XP_018504231.2. **(B)** Phylogenetic analysis of *PbMYB5* with different species. **(C)** Expression of the *PbMYB5* gene in various tissues. **(D)** Expression of the *PbMYB5* gene in the pericarp was measured at various developmental stages. **(E)** Subcellular localization of *PbMYB5*. *p<0.05, **p<0.01.

The transcriptome of ‘Red Zaosu’ pear epidermis was sequenced in the red region (RS) and in the red-yellow-green gradient region (GS) at 65, 85 and 105 d after anthesis, and KEGG and GO enrichment analyses showed that differentially differentiated genes were enriched in the phenylpropanoid pathway, with one of the genes 2459, having a significantly higher abundance in the RS than in the GS region at 65 d after anthesis and no significant difference in its abundance at 85 and 105 d (**Supplementary Figure S1**). By searching the GDR database, we found that the sequence of gene 2459 was highly similar to that of rna3106-v1.1-pbr and rna18247-v1.1-pbr in white pear, located on chromosome 3 and chromosome 11, with amino acid sequence lengths of 373 aa and 375 aa, respectively. Further sequence comparison showed that the sequence of gene 2459 had the highest homology with rna3106-v1.1-pbr (**Supplementary Figure S2**). The gene was therefore cloned from the peel of the ‘Red Zaosu’ pear and compared with the NCBI database, and it was found that several genes with high sequence similarity in different species belonged to the MYB family. Subsequent amino acid sequence conservation analyses showed that the ‘Red Zaosu’ pear skin gene 2459, like other MYB genes, has a complete R2R3 conserved structural domain (**Figure 1A**). Furthermore, a phylogenetic tree demonstrated that this gene was closely related to the predicted transcription repressor factor *MYB5* in ‘Hongxiangsu’ pear and *MdMYB5* in apple, support level of up to 99 (**Figure 1B**). Consequently, it was designated *PbMYB5*. Bioinformatics analysis revealed that the CDS sequence of *PbMYB5* was 1122 bp long and encoded a 373 amino acid protein. The amino acid sequence contained a highly conserved bHLH interaction motif (DLx2Rx3Lx6Lx3R) in the R3 structural domain, and no anthocyanin characterization motifs (ANDV and KPRPRS/TF) or inhibition motifs (LxLxL and TLLLFR) were found (Stracke et al., 2001). *PbMYB5* belongs to a remote clade with *PbMYB1*, *PbMYB10*, and *PbMYB114*. Quantitative gene expression analysis revealed that *PbMYB5* expression was higher in the leaves, buds, and flowers (**Figure 1D**). In the pericarp at different developmental stages, the highest expression of *PbMYB5* was observed at 45 DAF by 105 and 115 DAF (**Figure 1C**). The subcellular localisation analysis revealed that *PbMYB5* was present in both the nucleus and cytoplasm (**Figure 1E**).

### 3.2 *PbMYB5* inhibits lignin and PAs synthesis and promotes anthocyanin synthesis in pear pericarp

**Figure 2:**
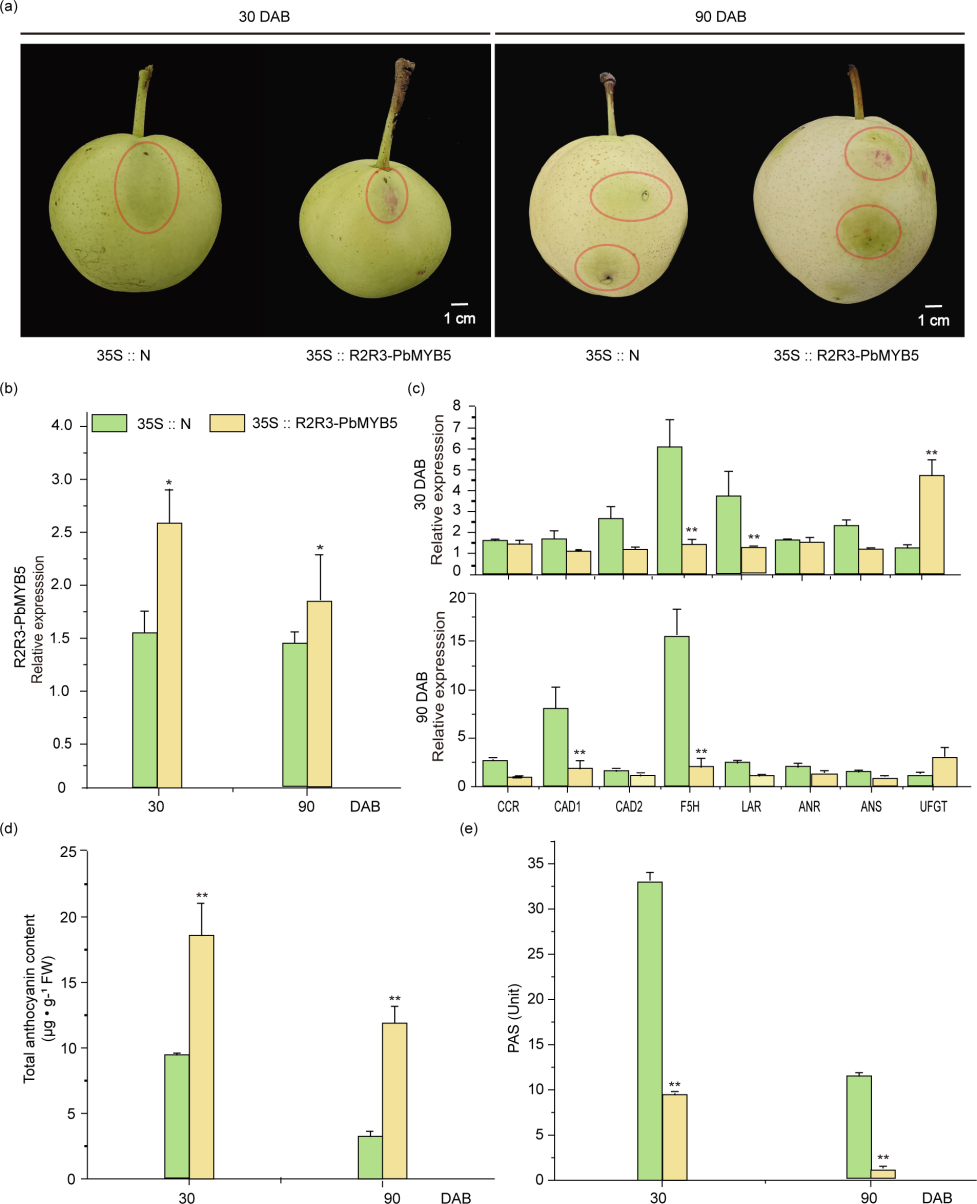
Phenotypes and associated gene expression after 35S::PbMYB5 injection in ‘Zaosu’ pears at different time points. **(A)** Overexpression of *PbMYB5* at different times and transient injection into ‘Zaosu’ pear fruit. The area where the injection is to be administered is indicated by the circle. **(B)** Lignin, PA and anthocyanin-related gene expression after overexpression of *PbMYB5*. **(C)** Expression of lignin, PAs and anthocyanin-related genes after overexpression of *PbMYB5*. **(D)** Anthocyanins after overexpression of *PbMYB5*. **(E)** PAs after overexpression of *PbMYB5*. *p<0.05, **p<0.01.

The 35S::*PbMYB5* overexpression vector and 35S::N empty vector were transiently injected into *‘*Zaosu*’* pear fruits using Agrobacterium transformation. Injections were administered 30 and 90 d after bagging, respectively. After one week of exposure, significant red accumulations were observed in both injected 35S::N. After one week of treatment, significant red accumulation was observed in all regions injected with 35S::*PbMYB* ***5***, whereas no red color accumulation was observed after 35S::N injection (**Figure 2A**). Examination of the epidermis injected with 35S::*PbMYB5* and 35S::N vectors revealed that 35S::*PbMYB5* increased the expression of *PbMYB5*. Expression was higher at 30 d after bagging than at 90 d (**Figure 2B**). Additionally, the expression of lignin synthesis genes *PbCAD1* and *PbF5H*, as well as the PAs synthesis gene *PbLAR*, was significantly lower than that of 35S::N during both stages. Conversely, after the injection of 35S::*PbMYB5*, the expression of the *PbUFGT* was significantly higher than that of 35S::N (**Figure 2C**). The results of the total anthocyanin and PAs content of the injected pericarp revealed that the anthocyanin content was higher than 35S::N and the PAs content was lower than 35S::N after injection of 35S::*PbMYB5* at 30 and 90 DAB, respectively (**Figure 2D, E**).

### 3.3 RNAi of *PbMYB5* promotes lignin and PA synthesis while suppressing anthocyanin synthesis in the pericarp of ‘Red Zaosu’

**Figure 3:**
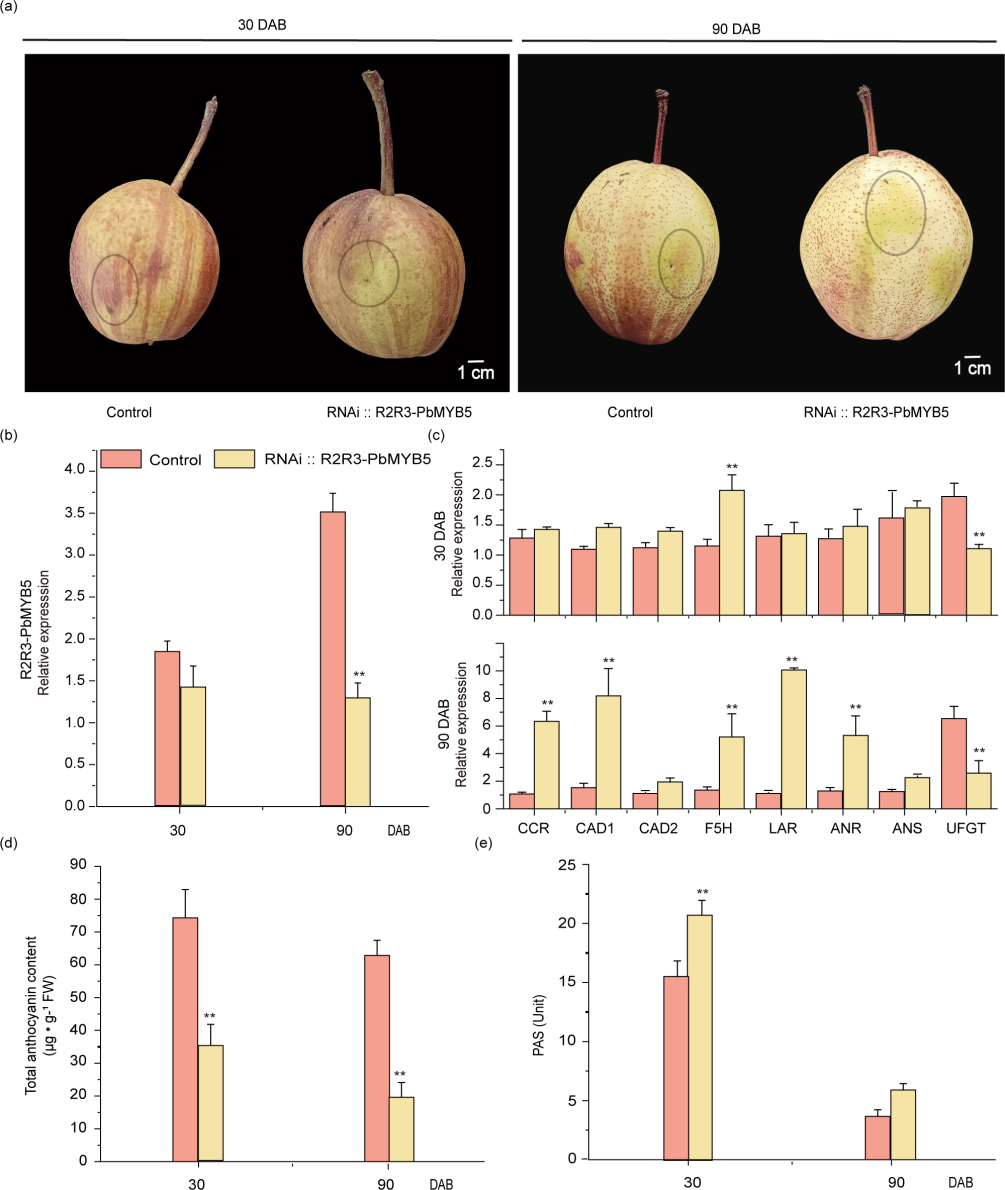
Phenotypes and associated gene expression after RNAi::*PbMYB5* injection in ‘RedZaosu’ pears at different time points. **(A)** Silencing of *PbMYB5* at different times for transient injection into ‘Red Zaosu’ pear fruit. The area where the injection is to be administered is indicated by the circle. **(B)** Gene expression after silencing *PbMYB5*. **(C)** Expression of lignin, PAs and anthocyanin-related genes after silencing of *PbMYB5*. **(D)** Anthocyanins content after silencing *PbMYB5*. **(E)** PAs after silencing *PbMYB5*. *p<0.05, **p<0.01.

The *PbMYB5* silencing vector (RNAi::*PbMYB5*) and the silencing null vector (RNAi::N) were transiently injected using *Agrobacterium* transformation into *‘*Red Zaosu*’* fruits at 30 and 90 d after bagging, respectively. After one week of exposure treatment, it was observed that none of the injected areas showed significant red accumulation after injection of RNAi::*PbMYB* ***5***. There was no significant red accumulation in any of the injected areas and no inhibition of red accumulation in the vicinity of the RNAi::N injection (**Figure 3A**). Examination of the epidermis after RNAi::*PbMYB5* and RNAi::N injections revealed that both RNAi::*PbMYB5* phases inhibited *PbMYB5* expression compared to RNAi::N (**Figure 3B**). Moreover, expression of lignin synthesis genes (*PbCAD1*, *PbF5H*) and PAs genes (*PbLAR* and *PbANR*) was higher than that of 35S::N, while the expression of anthocyanidin synthesis and transporter gene *PbUFGT* was significantly lower than that of 35S::N, after injection of RNAi::*PbMYB5* (**Figure 3C**). Results of the total anthocyanin and PAs content of the injected pericarp revealed that the anthocyanin content was lower than that of RNAi::N and the PA**s** content was higher than 35S::N after injection of RNAi::*PbMYB5* at 30 and 90 d after bagging, respectively (**Figures 3D, E**).

### 3.4 Transformation of *PbMYB5* and its effect on flavonoids in the pericarp of ‘Zaosu’ and ‘Red Zaosu’ pear during wound healing

**Figure 4:**
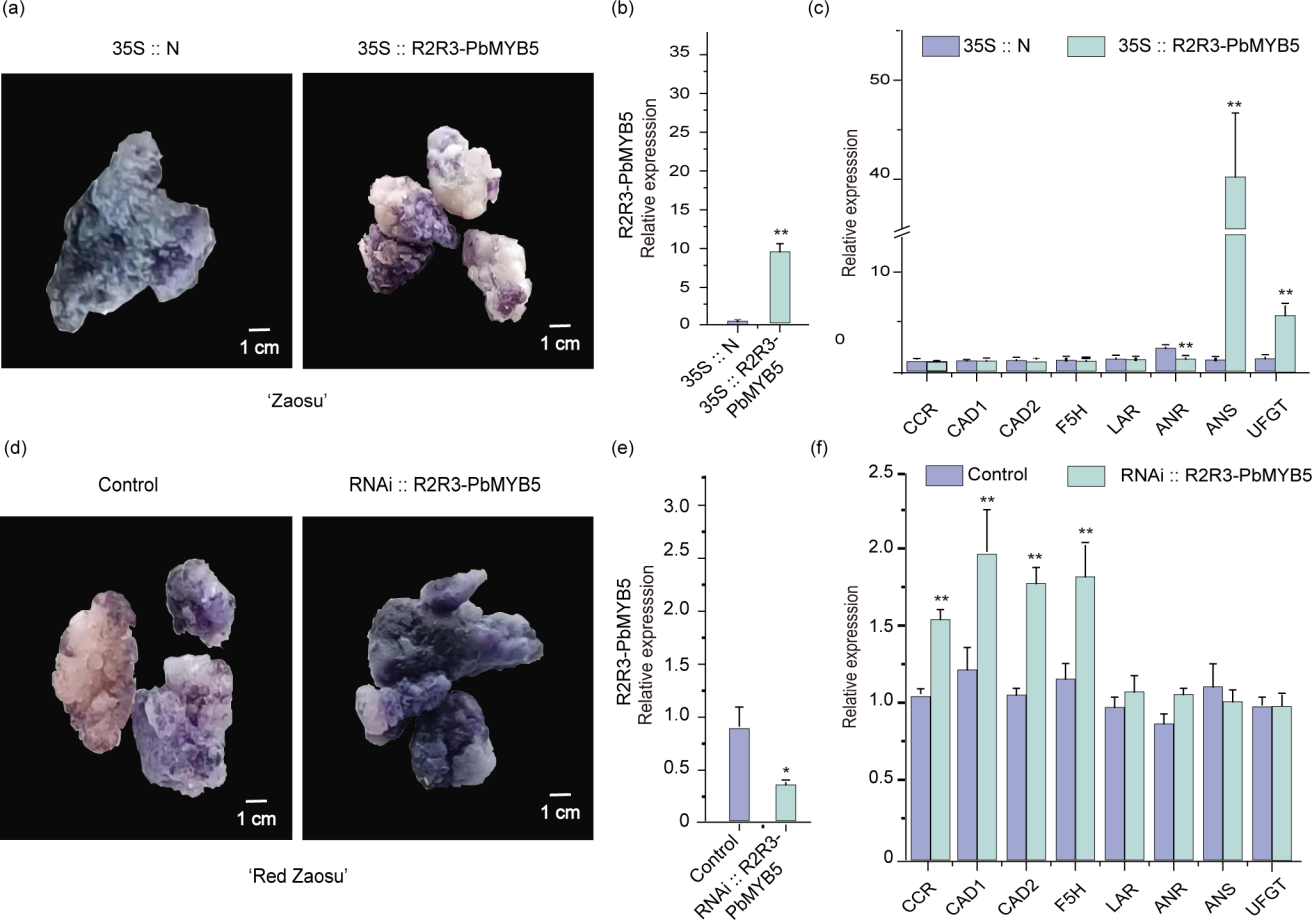
*PbMYB5* transformation of *‘*Zaosu*’* and *‘*Red Zaosu*’* pears, healing, and DMACA staining. **(A)** *PbMYB5* overexpression, ‘Zaosu’ pear healing DMACA staining phenotype. **(B)** Gene expression after *PbMYB5* overexpression. **(C)** Lignin, PAs, and anthocyanin-related gene expression after *PbMYB5* overexpression. **(D)** Silencing *PbMYB5*, *‘*Red Zaosu*’* pear healing DMACA staining phenotype. **(E)** Gene expression after silencing *PbMYB5*, **(F)** Lignin, PAs, and anthocyanin-related gene expression after silencing *PbMYB5*. *p < 0.05, **p < 0.01.

The *PbMYB5* overexpression vector (35S::*PbMYB5*) and the blank control overexpression vector (35S::N) were transformed into *‘*Zaosu*’* pear healing tissues. The positive healing tissue analysis revealed that 35S::*PbMYB5* promoted *PbMYB5* expression comparedto 35S::N (**Figure 4B**). Moreover, DMACA staining revealed that 35S::*PbMYB5* suppressed the expression of proanthocyanidins (**Figure 4A**) and related genes *PbLAR* and *PbANR*, while promoted the expression of *PbANS* and *PbUFGT* (**Figure 4C**). The *PbMYB5* silencing vector (RNAi::*PbMYB5*) and blank control silencing vector (RNAi::N) were transformed into ‘Red Zaosu’ pear healing tissues. Analysis of positive healing tissues showed that RNAi::*PbMYB5* was able to repress *PbMYB5* expression compared to control. (**Figure 4E**). Moreover, DMACA staining revealed that RNAi::*PbMYB5* promoted the expression of PAs (**Figure 4D**) and related genes *PbLAR* and *PbANR*, in addition to promoting the expression of lignin synthesis genes *PbCCR*, *PbCAD1*, *PbCAD2*, and *PbF5H*, and reducing the expression of anthocyanin synthesis gene *PbANS* (the difference was not statistically significant) (**Figure 4F**).

### 3.5 *PbMYB5* forms a novel complex with *bHLH3/bHLH33* for regulation of flavonoid metabolism

**Figure 5:**
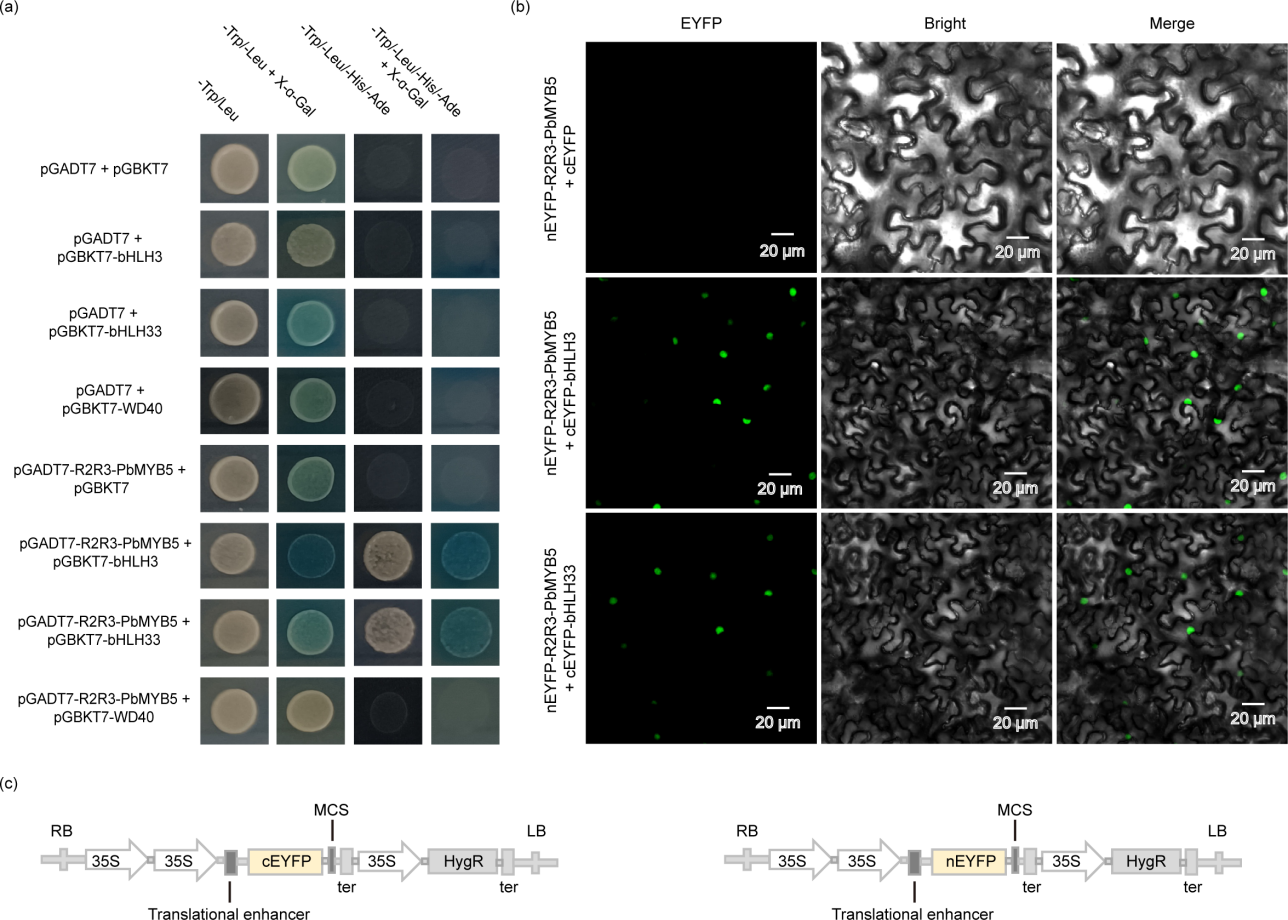
Validation of Yeast two-hybrid and BiFC interactions. **(A)** *PbMYB5*, *bHLH*, *WD40* yeast two-hybrid validation. **(B)** BiFC experimental validation. **(C)** Schematic representation of BiFC vector expression pattern.

The yeast two-hybrid results (**Figure 5**) revealed that *PbMYB5* can bind to *PbHLH3/PbHLH33* and does not directly interact with *PbWD40*. The BiFC assay revealed that *PbMYB5* interacts with *PbbHLH3/PbbHLH33* in the nuclei of tobacco cells. This suggests that *PbMYB5* forms a functional complex with *PbbHLH3/PbbHLH33* and forms an MYB-bHLH-WD40 (MBW) complex with the stabilizing protein *WD40*, which in turn regulates the structural genes involved in the anthocyanin pathway and regulates the metabolic flux and accumulation of anthocyanins and PAs.

### 3.6 *PbMYB5* has been demonstrated to bind to the *PbANS* promoter, thereby regulating anthocyanin metabolismPbMYB5

**Figure 6:**
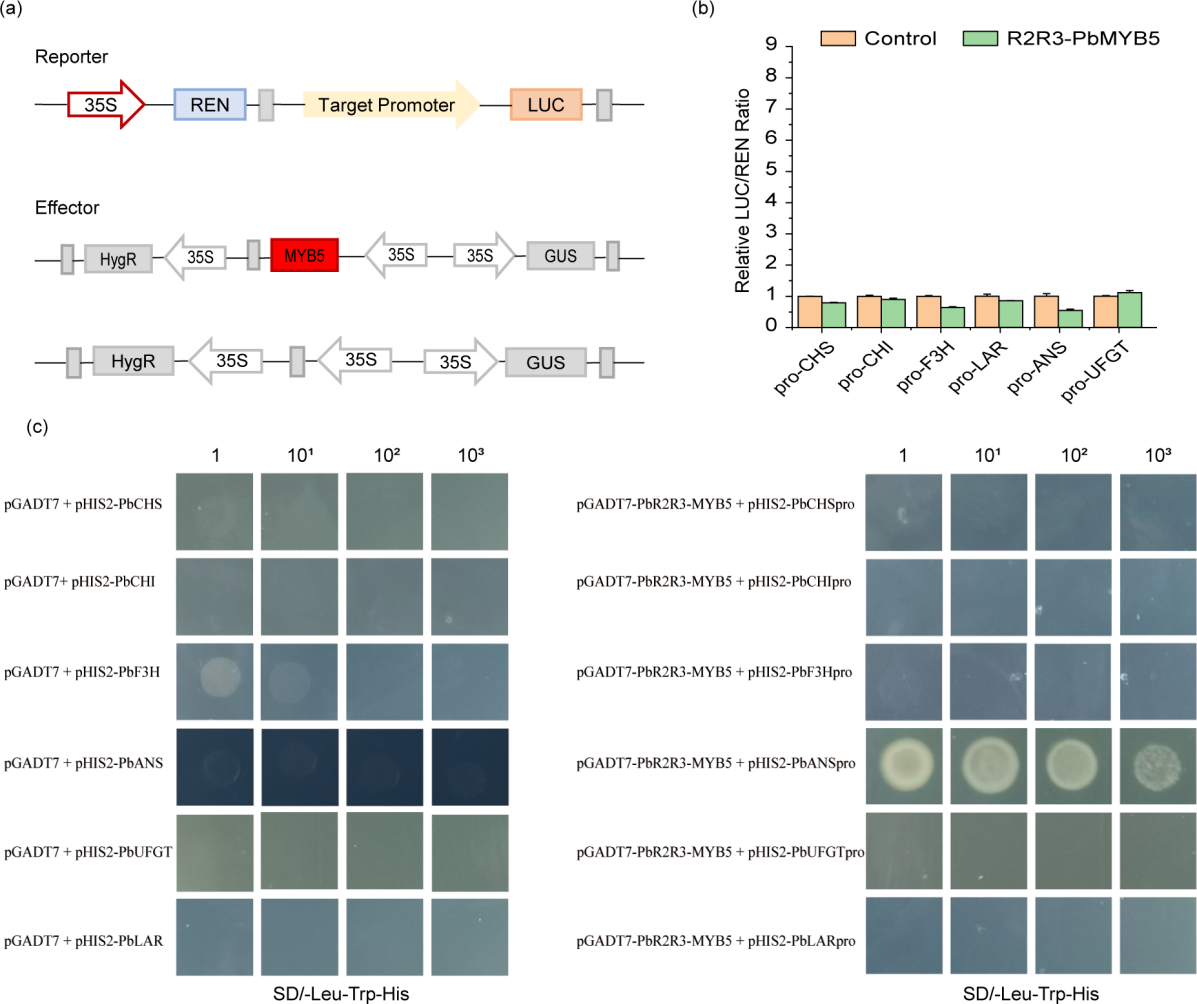
Complementary validation of the dual luciferase assay **(A)** Schematic representation of the *PbMYB5* dual luciferase. **(B)** Validation of *PbMYB5* interactions with the promoters of flavonoid pathway structural genes. **(C)** The objective of this study was to validate the interaction between *PbMYB5* and the promoters of structural genes involved in flavonoid metabolism using a yeast one-hybrid system. 10X represents the number of dilutions of the bacterial solution.

**Figure 7:**
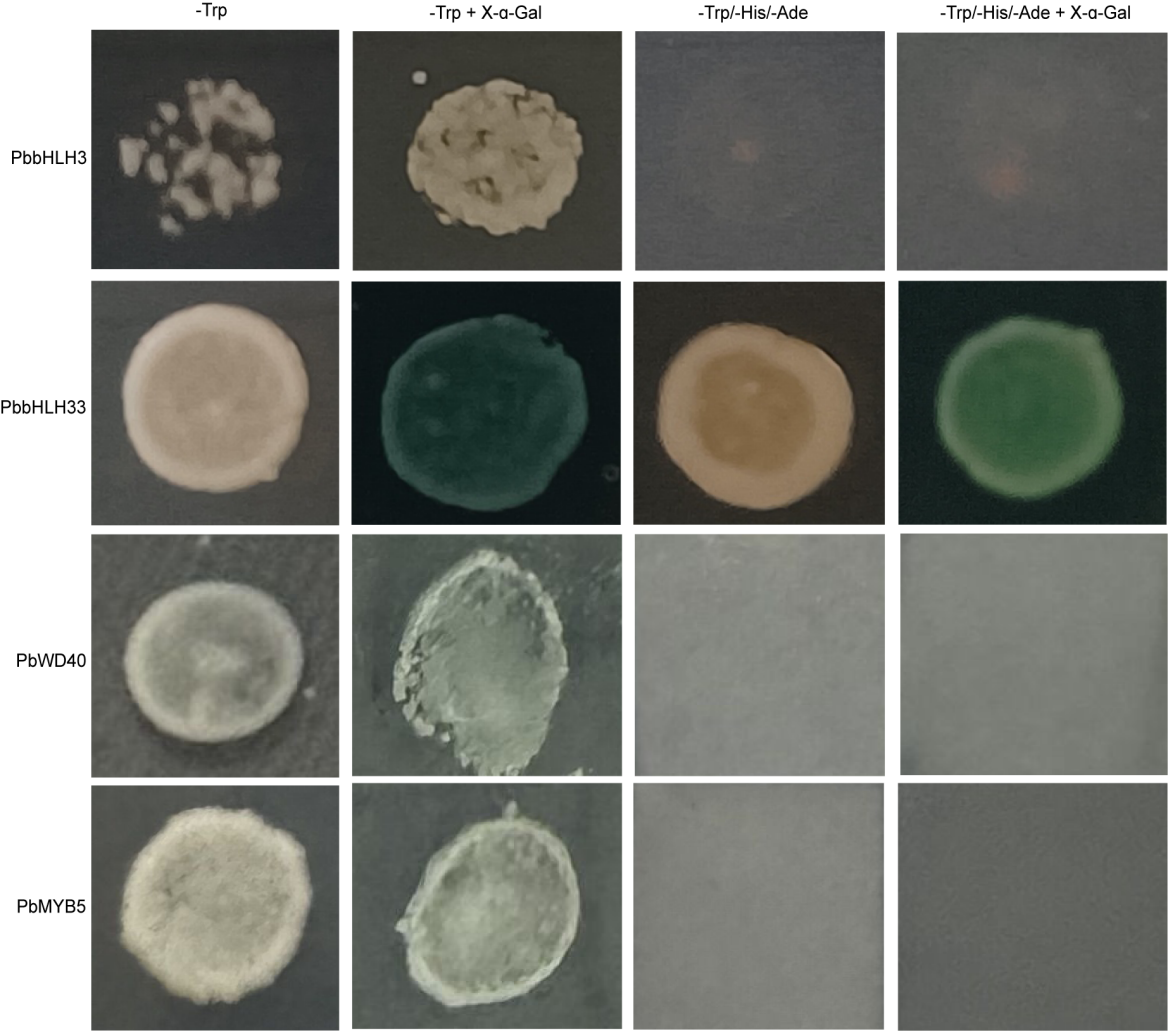
Verification of yeast self-activating activity.

The Dual luciferase assay showed that *PbMYB5* was not able to effectively activate the promoter activity of structural enzyme genes involved in the anthocyanin synthesis pathway (**Figures 6A, B**). The interaction between *PbMYB5* and *pro-PbANS* was observed in a yeast one-hybrid assay (**Figure 6C**), suggesting that *PbMYB5* may lack transcriptional activation abilities. To further support this idea, yeast self-activation assays were performed on *PbMYB5*, *bHLH3/33*, and *WD40*. It was found that, unlike *bHLH33*, *PbMYB5*, *bHLH3*, and *WD40* did not exhibit self-activating properties (**Figure 7**).

## 4 Discussion

### 4.1 *PbMYB5* affects pericarp coloration by regulating lignin and anthocyanoside metabolism

In the *Pyrus bretschneideri* genome (SAMN01797448 and SAMN12751541), *PbMYB5* was predicted to be a transcriptional repressor. In this study, *PbMYB5* was overexpressed in *‘*Zaosu*’* pear fruits and healing wounds and was found to promote reddening of the *‘*Zaosu*’* pear epidermis, promote anthocyanin accumulation, and regulate of PAs synthesis-related gene expression. Silencing *the PbMYB5* gene in *‘*Red Zaosu*’* pear fruits and healing wounds inhibited reddening of the *‘*Red Zaosu*’* pear epidermis, suppressed anthocyanin accumulation. The *PbMYB5* transcription factor promoted the accumulation of anthocyanins and regulated the accumulation of PAs in the pear epidermis, promoting the coloration of the pear epidermis. There are ongoing and well-defined studies on anthocyanin synthesis and metabolic regulation of fruit coloration. However, relatively little research has been done on transcriptional regulators that regulate crosstalk between different secondary metabolic pathways (e.g., anthocyanin, PAs, and lignin pathways). Photoinduction of apple pericarp promotes anthocyanin biosynthesis while reducing lignin production, indicating a negative correlation between anthocyanins and lignin and competition for metabolic flow (Hu et al., 2021). *R2R3-MYB6* promotes the accumulation of anthocyanins and PAs and inhibits secondary metabolites like lignin during the growth and development of poplar trees (Wang et al., 2019a). The results of this study revealed that overexpression of *PbMYB5* significantly increased the accumulation of red phenotypes in pear pericarp, decreasing the expression of the key enzyme genes for lignin synthesis, *CCR*, *CAD1*, and *F5H* and increasing the expression of the anthocyanin synthesis and transporter gene *UFGT*. Contrarily, silencing *PbMYB5* prevented the accumulation of red phenotype in the pear pericarp. These results are consistent with those of *MdMYB1*, which promoted anthocyanin accumulation and inhibited lignin synthesis (Zhou et al., 2017).

*VmMYBA1* and *VmMYBA2* upregulated the expression of genes involved in the anthocyanin and PAs pathways in blueberries. Overexpression of *MYB165* and *MYB194* inhibited PAs and anthocyanin synthesis in poplar and regulated the synthesis and metabolism of other phenolic substances (Ma et al., 2018). Overexpression of *VvMYC1* induced the accumulation of anthocyanins and PAs in grape berries. Furthermore, *VvMYB5a* overexpression increased the metabolism of anthocyanins, PAs, and lignin; *VvMYB5b* can induce the accumulation of anthocyanins and PAs derivatives, revealing a spatiotemporal specificity of *VvMYB5a* and *VvMYB5b* in regulating the accumulation of lignin, anthocyanins and PAs in grapevine fruits (Deluc et al., 2006, 2008). *The R2R3-FaMYB5* TF regulates anthocyanin and PAs metabolism in strawberry fruit and transgenic tissues. The results of this study revealed that *PbMYB5* expression was higher in pear fruits at 45 d after anthesis than at 105 and 115 d after anthesis and lower at 65 and 85 d after anthesis than at other times. These results are similar to those observed for grapes. *PbMYB5* overexpression increased anthocyanin synthesis in pear pericarp, consistent with the results of *MYB5* in strawberries and grapes. Nevertheless, in this study, *PbMYB5* overexpression resulted in the suppression of lignin and PAs synthesis gene expression in pear pericarp, and a significant inhibition of PA synthesis in pear pericarp and healing tissues. These results revealed that *PbMYB5* regulates the fluctuations of metabolic fluxes of different metabolic pathways and can affect pear skin color formation through metabolic flux competition.

### 4.2 *PbMYB5* binds *bHLH3/bHLH33* to form the MBW complex for the regulation of anthocyanin accumulation

Flavonoid pathways are generally influenced by the spatiotemporal expression specificity of their structural genes and, to a lesser extent, by regulation of the MBW complex (Gonzalez et al., 2008, 2010; Pu et al., 2021). In Arabidopsis, *MYB113/114* forms a complex with *TTG1* and *bHLH* to regulate the anthocyanin synthesis pathway in seedlings, whereas *TT2-TT8-TTG1* regulates PAs in seeds (Gonzalez et al., 2008; Xu et al., 2013). *R2R3-FaMYB5*, *FaEGL3*, *and FaLWD1/FaLWD1-like* may form a new MBW complex that regulates flavonoid metabolism in strawberries, *FaMYB9/FaMYB11-FabHLH3-FaTTG1* regulates the PAs pathway in fruits, and *FaMYB10-FabHLH3/FabHLH33-FaTTG1* may regulate anthocyanin metabolism (Jiang et al., 2023). In apples, *MdHB1* regulates anthocyanin synthesis by restricting *MdMYB10*, *MdbHLH3*, and *MdTTG1* to the cytoplasm, indirectly repressing the transcription of *MdDFR* and *MdUFGT* (Jiang et al., 2017). A ternary complex formed by *PyMYB10*, *PybHLH*, and *PyWD40* transcription factors regulates anthocyanin biosynthesis and accumulation in the *‘*Yunnan Hong*’* Pear (Cui et al., 2021). *PpMYB114* and *PpbHLH3* promote *PpMYB114*-induced *PpUFGT* expression and red color accumulation; anthocyanin accumulation is transcriptionally regulated by the MYB-bHLH-WD40 complex (Ni et al., 2019). In this study, we discovered that *PbMYB5* could interact with *bHLH3/bHLH33* but not with *WD40*. This result is consistent with previous reports that MYB transcription factors interact with *bHLH3* to form MYB-bHLH and *that WD40* acts as a stabilizing protein that interacts with *bHLH*, forming the *MYB-bHLH-WD* complex (Alabd et al., 2022; Cheng et al., 2014; Ni et al., 2021; Wang et al., 2019b; Ye et al., 2016). The spatiotemporal specificity of *PbMYB5* contributes to the dynamics of *PbMYB5-bHLH3/bHLH33-WD40* complex, which in turn inhibits the expression of lignogenic anthocyanin genes, reduces the flux of related metabolites, promotes the expression of anthocyanin-related genes and accumulation of anthocyanin glycosides, and influences the coloration of pear skin.

### 4.3 *PbMYB5* induces the promoter activity of the anthocyanin structural gene and regulates the metabolism of anthocyanins

**Figure 8:**
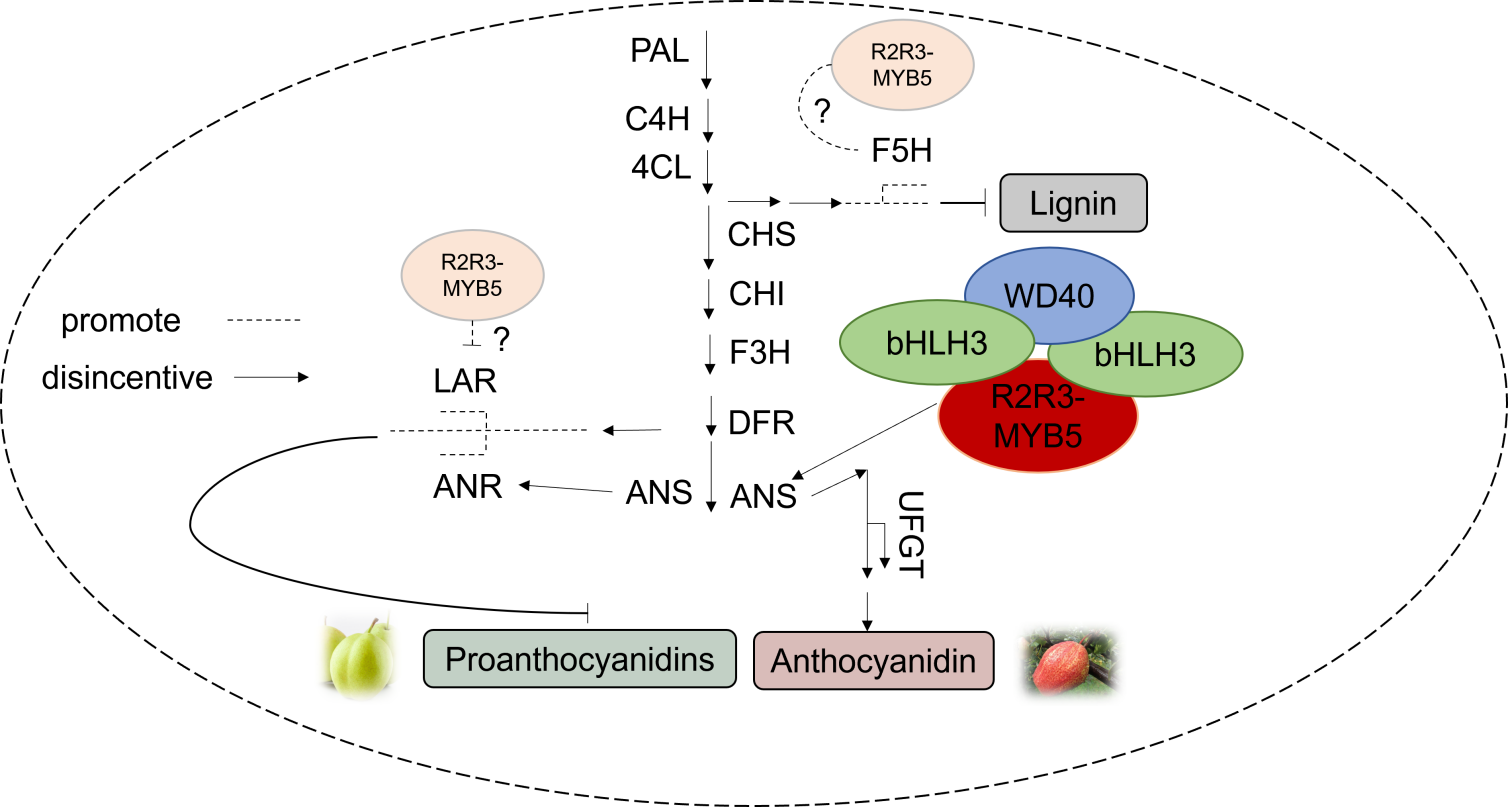
Pattern of *PbMYB5* regulation of different pathways in pear pericarp.

Changes in the expression of anthocyanin structural genes are controlled by transcriptional regulators. In strawberries, *R2R3-FaMYB5* and *FaMYB10* both promote the expression of most of the structural genes involved in the flavonoid biosynthetic pathway (*PAL*, *C4H*, *4CL-2*, and *F3’H*), and *R2R3-FaMYB5* can specifically regulate the PAs-associated gene *LAR* (Jiang et al., 2023). *PpMYB17* positively regulates flavonoid biosynthesis in pear fruit by activating *PpCHS*, *PpCHI*, *PpF3H*, and *PpFLS* in the flavonoid biosynthetic pathway independently of the *bHLH* or *WD40* cofactors in the MBW complex (Premathilake et al., 2020). Our findings suggest that *PbMYB5* does not have inherent activation ability, but can specifically bind to the *PbANS* promoter to enhance anthocyanin production. It appears that *bHLH33* may form complexes that activate *PbMYB5* and promote *PbAN*S gene expression. The slight discrepancy in results compared to previous studies may be due to the direct regulation of *PbUFGT* by the *PbMYB5-bHLH-WD40* complex, as well as the transactivation of *PbANS* by *PbMYB5* with the assistance of *bHLH33*, resulting in increased anthocyanidin synthesis and disruption of *PbANS* Regulation (**Figure 8**).

## 5 Conclusions

The study results show that *PbMYB5* plays a role in enhancing anthocyanin accumulation and controlling the expression of genes related to lignin and PAs synthesis in pear skin. *PbMYB5* forms a complex with *bHLH3/bHLH33-WD40*, which promotes anthocyanin accumulation in pears. Additionally, *PbMYB5* can combine with *PbANS*, activating its promoter with *bHLH33*. These findings provide insights into metabolite regulation in pear skin and offer new strategies for molecular breeding and variety enhancement.

## Data Availability

The original contributions presented in the study are included in the article/**Supplementary Material**. Further inquiries can be directed to the corresponding authors.
