# Supplementary figures and images for: PbMYB5 transcription factor plays a role in regulating anthocyanin biosynthesis in pear (Pyrus bretschneideri Rehd) skin
Source: Front Plant Sci. 2025 Jan 14;15:1492384. doi: 10.3389/fpls.2024.1492384 (PMC11772430; doi:10.3389/fpls.2024.1492384)

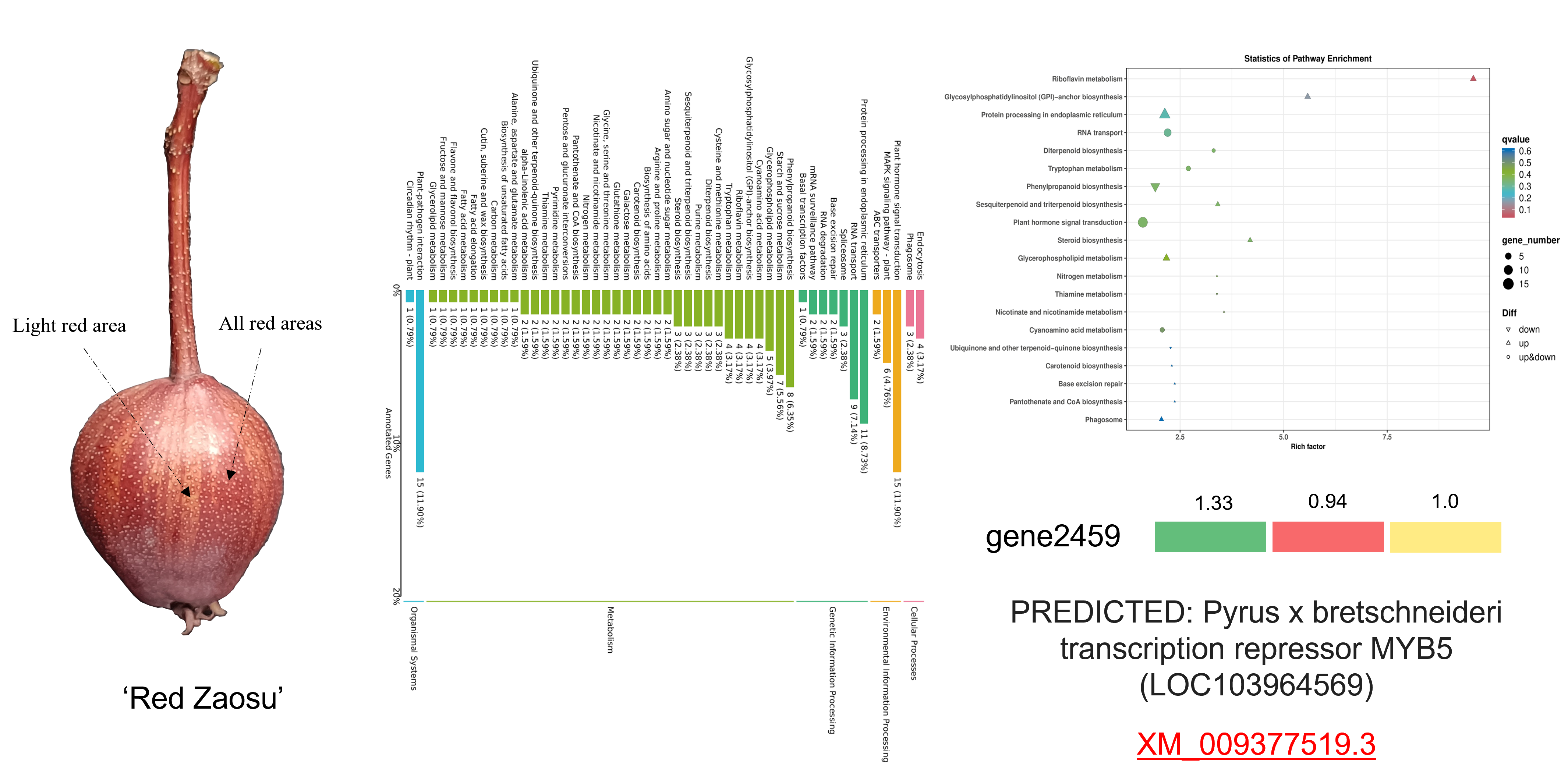

Supplement: Supplementary file 1 [file Image1.tif]
