# Supplementary material for: PbMYB5 transcription factor plays a role in regulating anthocyanin biosynthesis in pear (Pyrus bretschneideri Rehd) skin
Source: Front Plant Sci. 2025 Jan 14;15:1492384. doi: 10.3389/fpls.2024.1492384 (PMC11772430; doi:10.3389/fpls.2024.1492384)
Supplement: Supplementary file 4 [file Table3.docx]

>gene2459 rna3107 (CDS, Sequences from transcriptome sequencing)

ATGAGGAACCCCCCGTCGTCGTCGAAAGCAACAGCAGCAGCAAGTGCTACGATGACAACAGCATCGACGTCGTCGAGTAAGGCGGGGATTGCCGGAGGGAGTAAGACGCCGTGTTGTGTAAAGGTGGGTTTGAAGAGGGGGCCGTGGACTCCTGAAGAGGACGAGCTGCTGGCCAATTACATCAAGAAAGAAGGTGAAGGACGGTGGCGGACCCTCCCCAAGCAGGCTGGGTTGCTCCGCTGCGGAAAAAGCTGTCGCCTCCGCTGGATGAACTACCTCCGCCCTTCCGTTAAGCGCGGCCAGATCGCCCCCGATGAAGAAGATCTCATTCTCCGCCTCCATCGCCTCCTCGGCAATCGGTGGTCTTTGATAGCTGGGAGGATTCCAGGTCGTACGGACAATGAGATAAAGAACTACTGGAACACACACCTGAGCAAGAAGCTGATAAGCCAAGGCATAGATCCCAGAACCCACAAGCCTCTCAATCCAGATCATCACTCTGCTGCTGCCGATGCTGATGTGGACAACACAAATAAATTAGTTGCTGCTGCTGCTTCTTTCAAGGCTAATACCCGGTTCTCAAACCCTAATCCTAGTCCTCCTCCTTCTGATCGTCTTGTCCATCAAGGAGCGGATCCCAGTATCAACGGTAATGATGGAAACATCGCAATTGATCATGATCTGGGTACTATAGTCCATAGCTGTGCAAACTTGATCACGTCCATTAACAATCCCGATGCTTCTTCTTCGGCCGCAGCAATGGGCACTTCGAGTTTAAGGACCAACAACAACAGCCAGGCTGGAGTACTACTTGGGGGAGGAGGAAATGAAGAGGACGAGGACATCAACTGTTGTGCGGACGACGTCTTCTCTTCGTTTCTGAATTCGTTGATCAACGAGGATCCATTTGCTGTACAACACCAATTGCAACAACAGGTACTGCACAATGGGAATGTTAGTACACACGCAGCTGGTGCTGGTTCCGACCACGTTCCTTTGATTTCTATGACTAGTGCTAGTACTATGGTGCCGTCAACATTTGGCTGGGACTCTGCTGTGCTCATGTCTTCTGCTTTCATCCAAAATGATCACCAGAGGGTTACTGATCAAACGGAGCAGTAG

>XM_009377519.3:374-1495 PREDICTED: Pyrus x bretschneideri transcription repressor MYB5 (LOC103964569), mRNA (NCBI Sequence Alignment)

ATGAGGAACCCCCCGTCGTCGTCGAAAGCAACAGCAGCAGCAAGTGCTACGATGACAACAGCATCGCCGTCGTCGAGTAAGGCGGGGATTGCCGGAGGGAGTAAGACGCCGTGTTGCGTAAAGGTGGGTTTGAAGAGGGGGCCGTGGACTCCCGAAGAGGACGAGCTGCTGGCCAATTACATCAAGAAAGAAGGTGAAGGACGGTGGCGGACCCTCCCTAAGCAGGCTGGGTTGCTCCGCTGCGGAAAAAGCTGTCGCCTCCGCTGGATGAACTACCTCCGCCCTTCCGTTAAGCGCGGCCAGATCGCCCCCGATGAAGAAGATCTCATTCTCCGCCTCCATCGCCTCCTCGGCAATCGGTGGTCTTTGATAGCTGGGAGGATTCCAGGTCGTACGGACAATGAGATAAAGAACTACTGGAACACACACCTGAGCAAGAAGCTGATAAGCCAAGGCATAGATCCCAGAACCCACAAGCCTCTCAATCCAGATCATCACTCTGCTGCTGCCGATGCTGATGTGGACAACACAAATAAATTAGTTGCTGCTGCTGCTTCTTTCAAGGCCAATACCCGGTTCTCAAACCCTAATCCTAGTCCTCCTCCTTCTGATCGTCTTGTCCATCAAGGAGCGGATCCCAGTATCAACGGTAATGATGGAAACATCGCAATTGATCATGATCTGGGTACTATAGTCCATAGCTGTGCAAACTTGATCACGTCCATTAACAATCCCGATGCTTCTTCTTCGGCCGCAGCAATGGGCACTTTGAGTTTAAGGACCAACAACAACAGCCACGCTGGAGTACTACCTGGGGGAGGAGGAAATGAAGAGGACGAGGACATCAACTGTTGTGCGGACGACGTCTTCTCTTCGTTTCTGAATTCGTTGATCAACGAGGATCCATTTGCTGTACAACACCAATTGCAACAACAGGTACTGCACAATGGGAATGTTAGTACACACGCAGCTGGTGCTGGTTCCGACCACGTTCCTTTGATTTCTATGACTAGTGCTAGTACTATGACGCCGTCAACATTTAGCTGGGACTCTGCTGTGCTCATGTCTTCTGCTTTCATCCAAAATGATCACCAGAGGGTTACTGATCAAACGGAGCAGTAG

>XM_068455403.1:430-1551 PREDICTED: Pyrus communis transcription repressor MYB5-like (LOC137716017), mRNA (NCBI Sequence Alignment)

ATGAGGAACCCCCCGTCGTCGTCGAAAGCAACAGCAGCAGCAAGTGCTACGATGACAACAGCATCGCCGTCGTCGAGTAAGGCGGGGATTGCCGGAGGGAGTAAGACGCCGTGTTGCGTAAAGGTGGGTTTGAAGAGGGGGCCGTGGACTCCCGAAGAGGACGAGCTGCTGGCCAATTACATCAAGAAAGAAGGTGAAGGACGGTGGCGGACCCTCCCTAAGCAGGCTGGGTTGCTCCGCTGCGGAAAAAGCTGTCGCCTCCGCTGGATGAACTACCTCCGCCCTTCCGTTAAGCGCGGCCAGATCGCCCCCGATGAAGAAGATCTCATTCTCCGCCTCCATCGCCTCCTCGGCAATCGGTGGTCTTTGATAGCTGGGAGGATTCCAGGTCGTACGGACAATGAGATAAAGAACTACTGGAACACACACCTGAGCAAGAAGCTGATAAGCCAAGGCATAGATCCCAGAACCCACAAGCCTCTCAATCCAGATCATCACTCTGCTGCTGCCGATGCTGATGTGGACAACACAAATAAATCAGTTGCTGCTGCTGCTTCTTTCAAGGCCAATACCCGGTTCTCAAACCCTAATCCTAGTCCTCCTCCTTCTGATCGTCTTGTCCATCAAGGAGCGGATCCCAGTATCAACGGTAATGATGGAAACATCGCAATTGATCATGATCTGGGTACTATAGTCCATAGCTGTGCAAACTTGATCACGTCCATTAACAATCCCGATGCTTCTTCTTCGGCCGCAGCAATGGGCACTTTGAGTTTAAGGACCAACAACAACAGCCACGCTGGAGTACTACTTGGGGGAGGAGGAAATGAAGAGGACGAGGACATCAACTGTTGTGCGGACGACGTCTTCTCTTCGTTTCTGAATTCGTTGATCAACGAGGATCCATTTGCTGTACAACACCAATTGCAACAACAGGTACTGCACAATGGGAATGTTAGTACACACGCAGCTGGTGCTGGTTCCGACCACGTTCCTTTGATTTCTATGACTAGTGCTAGTACTATGACGCCGTCAACATTTAGCTGGGACTCTGCTGTGCTCATGTCTTCTGCTTTCATCCAAAATGATCACCAGAGGGTTACTGATCAAACGGAGCAGTAG

>rna3106-v1.1-pbr chr3-v1.1-pbr chr3-v1.1-pbr:19302389..19305691 (- strand) class=mRNA length=3303 (GDR Sequence Alignment)

ATGAGGAACCCCCCGTCGTCGTCGaaagcaacagcagcagcaagtGCTACGATGACAACAGCATCGACGTCGTCGAGTAAGGCGGGGATTGCCGGAGGGAGTAAGACGCCGTGTTGTGTAAAGGTGGGTTTGAAGAGGGGGCCGTGGACTCCTGAAGAGGACGAGCTGCTGGCCAATTACATCAAGAAAGAAGGTGAAGGACGGTGGCGGACCCTCCCCAAGCAGGCTGGGTTGCTCCGCTGCGGAAAAAGCTGTCGCCTCCGCTGGATGAACTACCTCCGCCCTTCCGTTAAGCGCGGCCAGATCGCCCCCGATGAAGAAGATCTCATTCTCCGCCTCCATCGCCTCCTCGGCAATCGGTGGTCTTTGATAGCTGGGAGGATTCCAGGTCGTACGGACAATGAGATAAAGAACTACTGGAACACACACCTGAGCAAGAAGCTGATAAGCCAAGGCATAGATCCCAGAACCCACAAGCCTCTCAATCCAGATCATCACTCTGCTGCTGCCGATGCTgatgtggacaacacaaataaattagttgctgctgctgcttctttcAAGGCTAATACCCGGTTCTCAAACCCTAATCCTAGTCCTCCTCCTTCTGATCGTCTTGTCCATCAAGGAGCGGATCCCAGTATCAACGGTAATGATGGAAACATCGCAATTGATCATGATCTGGGTACTATAGTCCATAGCTGTGCAAACTTGATCACGTCCATTAACAATCCCGATGCTTCTTCTTCGGCCGCAGCAATGGGCACTTCGAGTTTAAGGACCAACAACAACAGCCAGGCTGGAGTACTACTTGGGGGAGGAGGAAATGAAGAGGACGAGGACATCAACTGTTGTGCGGACGACGTCTTCTCTTCGTTTCTGAATTCGTTGATCAACGAGGATCCATTTGCTGTACAACACCAATTGCAACAACAGGTACTGCACAATGGGAATGTTAGTACACACGCAGCTGGTGCTGGTTCCGACCACGTTCCTTTGATTTCTATGACTAGTGCTAGTACTATGGTGCCGTCAACATTTGGCTGGGACTCTGCTGTGCTCATGTCTTCTGCTTTCATCCAAAATGATCACCAGAGGGTTACTGATCAAACGGAGCAGTAG

>rna18247-v1.1-pbr chr11-v1.1-pbr chr11-v1.1-pbr:4978483..4980581 (- strand) class=mRNA length=2099 (GDR Sequence Alignment)

ATGAGGAACCCATCGCCTTCGTcgaaagcagcagcagcagcagcaagtgCTAAGATGCAAACGACGATAACAGCGTCGTCCTCGTCGAGCAAGGCGGCTGGGGTTGCTGGAGGGACCAAGACGCCGTGTTGCGCAAAGGTGGGTTTGAAGAGAGGGCCGTGGACTCCCGAAGAGGACGAGCTGCTGGCAAATTACATCAAGAAAGAAGGGGAGGGACGGTGGCGGACCCTTCCCAAGCGGGCTGGGTTGCTCCGATGCGGTAAGAGCTGCCGCCTCCGCTGGATGAACTATCTCCGCCCTTCCGTCAAGCGCGGCCAGATCGCCCCCGATGAAGAAGATCTCATCCTTCGCCTCCATCGCCTTCTGGGCAATCGGTGGTCTTTGATAGCTGGGAGGATTCCAGGCCGTACGGACAATGAGATAAAGAACTACTGGAACACACACCTGAGCAAGAAGCTGATAAACCAAGGCATAGATCCCAGAACCCACAAGCCTCTCAATCCAGATCATCACTCTGCTGCTGATGATGCTGACCTggacaacacaaacaaatcaACTGCTGTTGCTTCTTCTTCCAAAGCCAATGATCGGTtctcaaaccctaaccctagtcCCCCTTCTGATCGTCTTGTCCATAAAGAAGGGGATCCAAATAACAGCCGTAATGGTGGAAACATCGCAATTGATGATCATGATCAGGGCACTATAGTCCATGGCTATGCAAATATGATCACGTCCATCAACAATCCCGATGCTTCTTCTTCGGCCACGGCAACGGGTACTTTGAGTTTGAGGAGCAACAACAGCCACGGTGGAGTACTACTTGGGGGAGGAGGAAATGAAGAGGACGACGACATCAACTGTTGTGCGGACGACGTCTTCTCTTCGTTTCTGAATTCGTTGATCAATGAGGATCCATTTCATGGACAACACCAATTGCAACAAGTACTGCAGAATGGGAATGTGAGTGCACACGCAGCTGCTGCTGGTTCCGAGAACCTTCCTTTGATTACTATGACTGGTGCTAGTACTACGGCGCCATCAACATTTGGCTGGGAGTCTGCTGTGCTCATGTCTTCTGCTTTCATCCATAATGATCACCAAAGGGTTAATGATCCAACGGAGTAG

PbMYB5 ‘Red Zaosu’ (Sequences cloned from the pericarp of ‘Red Zaosu’ pear)

ATGAGGAACCCCCCGTCGTCGTCGAAAGCAACAGCAGCAGCAAGTGCTACGATGACAACAGCATCGCCGTCGTCGAGTAAGGCGGGGATTGCCGGAGGGAGTAAGACGCCGTGTTGCGTAAAGGTGGGTTTGAAGAGGGGGCCGTGGACTCCCGAAGAGGACGAGCTGCTGGCCAATTACATCAAGAAAGAAGGTGAAGGACGGTGGCGGACCCTCCCTAAGCAGGCTGGGTTGCTCCGCTGCGGAAAAAGCTGTCGCCTCCGCTGGATGAACTACCTCCGCCCTTCCGTTAAGCGCGGCCAGATCGCCCCCGATGAAGAAGATCTCATTCTCCGCCTCCATCGCCTCCTCGGCAATCGGTGGTCTTTGATAGCTGGGAGGATTCCAGGTCGTACGGACAATGAGATAAAGAACTACTGGAACACACACCTGAGCAAGAAGCTGATAAGCCAAGGCATAGATCCCAGAACCCACAAGCCTCTCAATCCAGATCATCACTCTGCTGCTGCCGATGCTGATGTGGACAACACAAATAAATCAGTTGCTGCTGCTGCTTCTTTCAAGGCCAATACCCGGTTCTCAAACCCTAATCCTAGTCCTCCTCCTTCTGATCGTCTTGTCCATCAAGGAGCGGATCCCAGTATCAACGGTAATGATGGAAACATCGCAATTGATCATGATCTGGGTACTATAGTCCATAGCTGTGCAAACTTGATCACGTCCATTAACAATCCCGATGCTTCTTCTTCGGCCGCAGCAATGGGCACTTTGAGTTTAAGGACCAACAACAACAGCCACGCTGGAGTACTACCTGGGGGAGGAGGAAATGAAGAGGACGAGGACATCAACTGTTGTGCGGACGACGTCTTCTCTTCGTTTCTGAATTCGTTGATCAACGAGGATCCATTTGCTGTACAACACCAATTGCAACAACAGGTACTGCACAATGGGAATGTTAGTACACACGCAGCTGGTGCTGGTTCCGACCACGTTCCTTTGATTTCTATGACTAGTGCTAGTACTATGACGCCGTCAACATTTAGCTGGGACTCTGCTGTGCTCATGTCTTCTGCTTTCATCCAAAATGATCACCAGAGGGTTACTGATCAAACGGAGCAGTAG


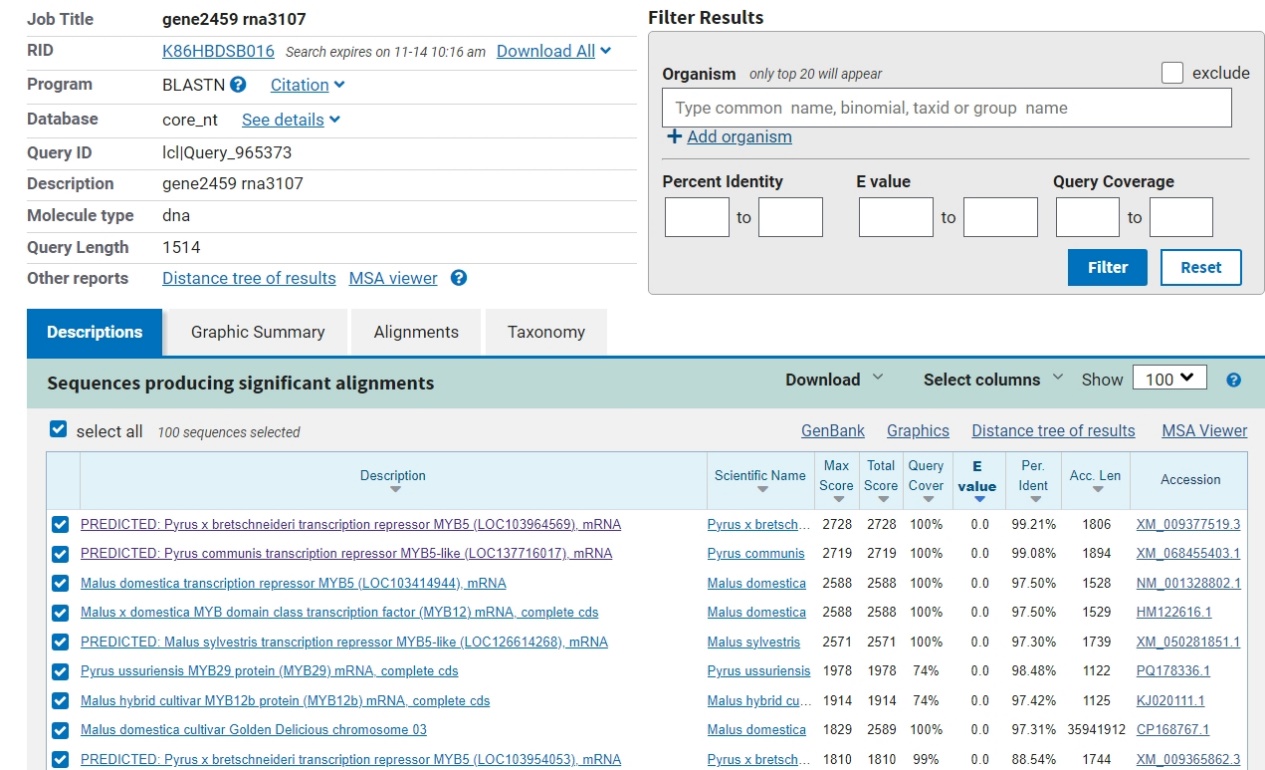


1. The nucleotide sequence of gene2459, obtained by transcriptome sequencing mining, was compared in the NCBI database and found to be highly similar to the sequence of Pyrus x bretschneideri transcription repressor MYB5 (LOC103964569).


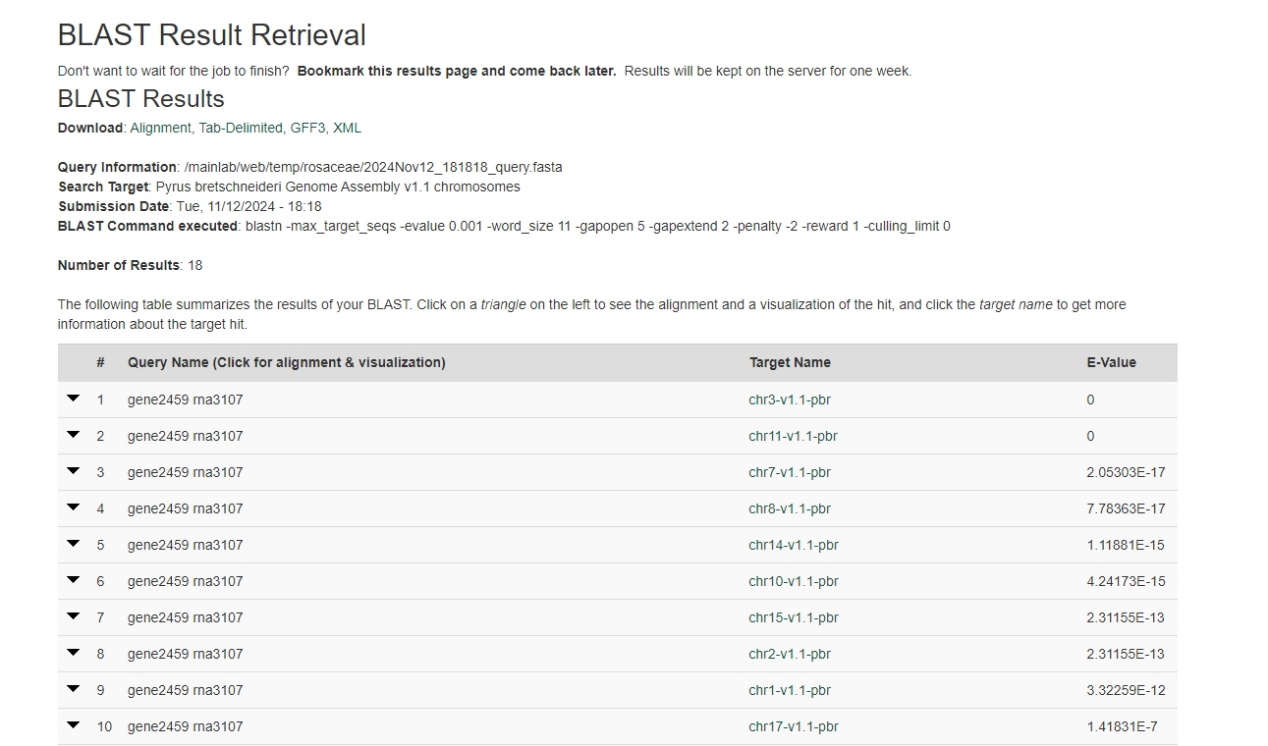


2.The nucleotide sequence of gene2459 obtained by transcriptome sequencing mining was further compared in the Rosaceae GDR database and found to be highly similar to the sequences of rna3106-v1.1-pbr and >rna18247-v1.1-pbr, with the highest similarity and the strongest homology in rna3106-v1.1-pbr.

Note:

GDR database:Pyrus bretschneideri ‘DangshanSuli’ Genome Assembly v1.1

NCBI database:Pyrus x bretschneideri cultivar:Hongxiangsu RefSeq Genome sequencing and assembly.The reference sequence (RefSeq) genome assembly is derived from the submitted GenBank assembly (see linked project PRJNA565550). Annotation provided on the RefSeq genomic records is based on NCBI annotation pipeline.


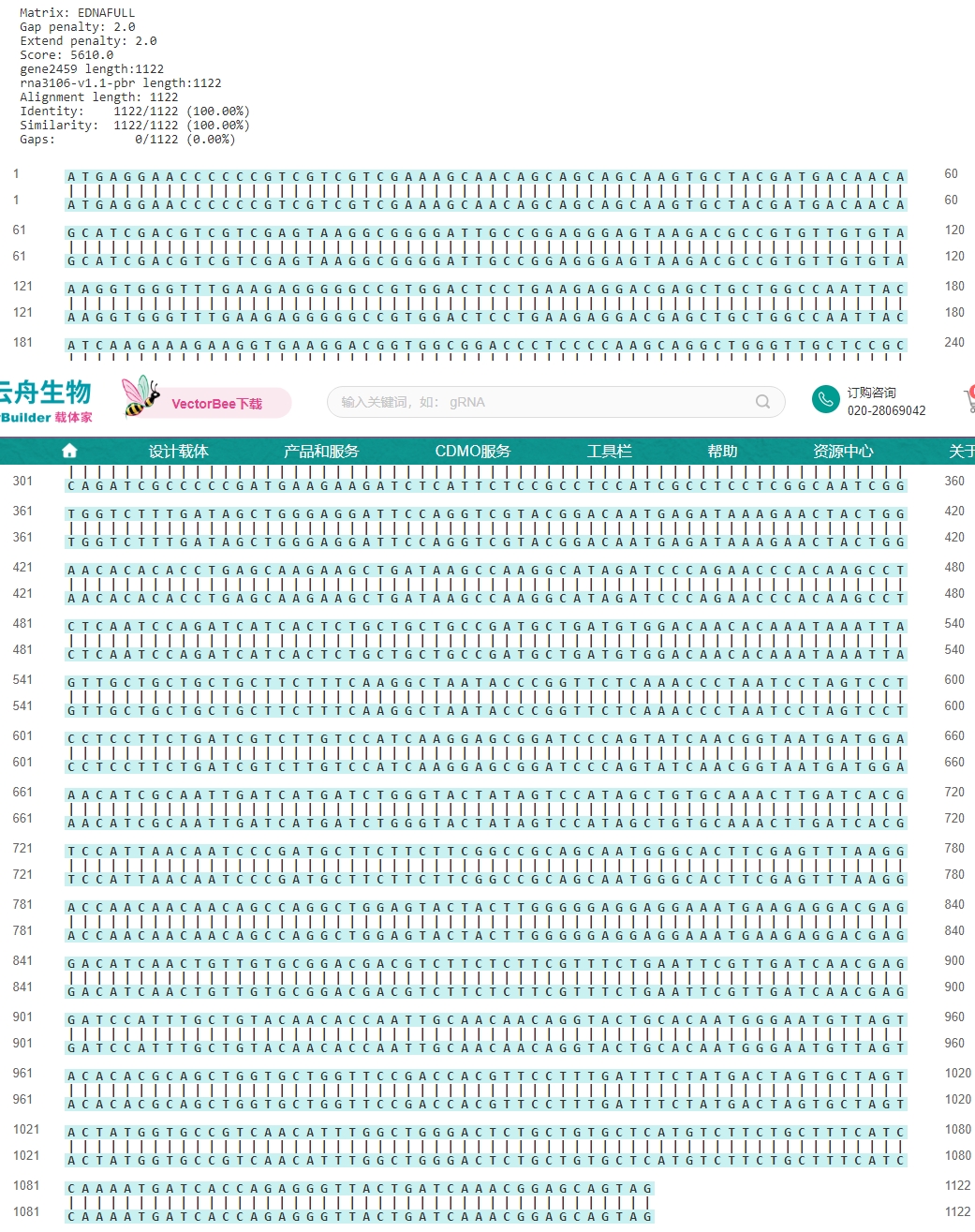

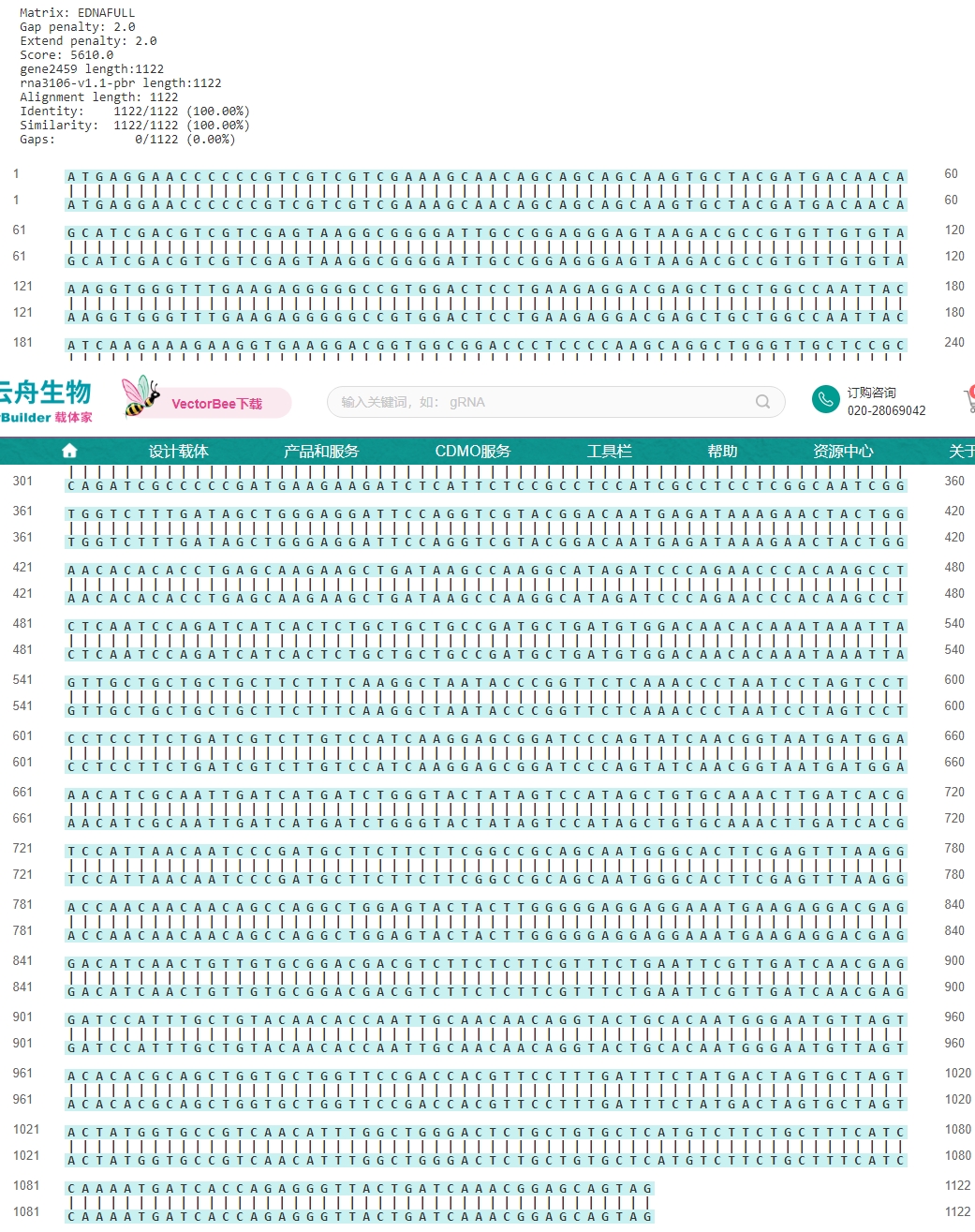


The results of the comparison of gene2459 with the CDS region of the sequence of rna3106-v1.1-pbr showed a sequence similarity of 100%.


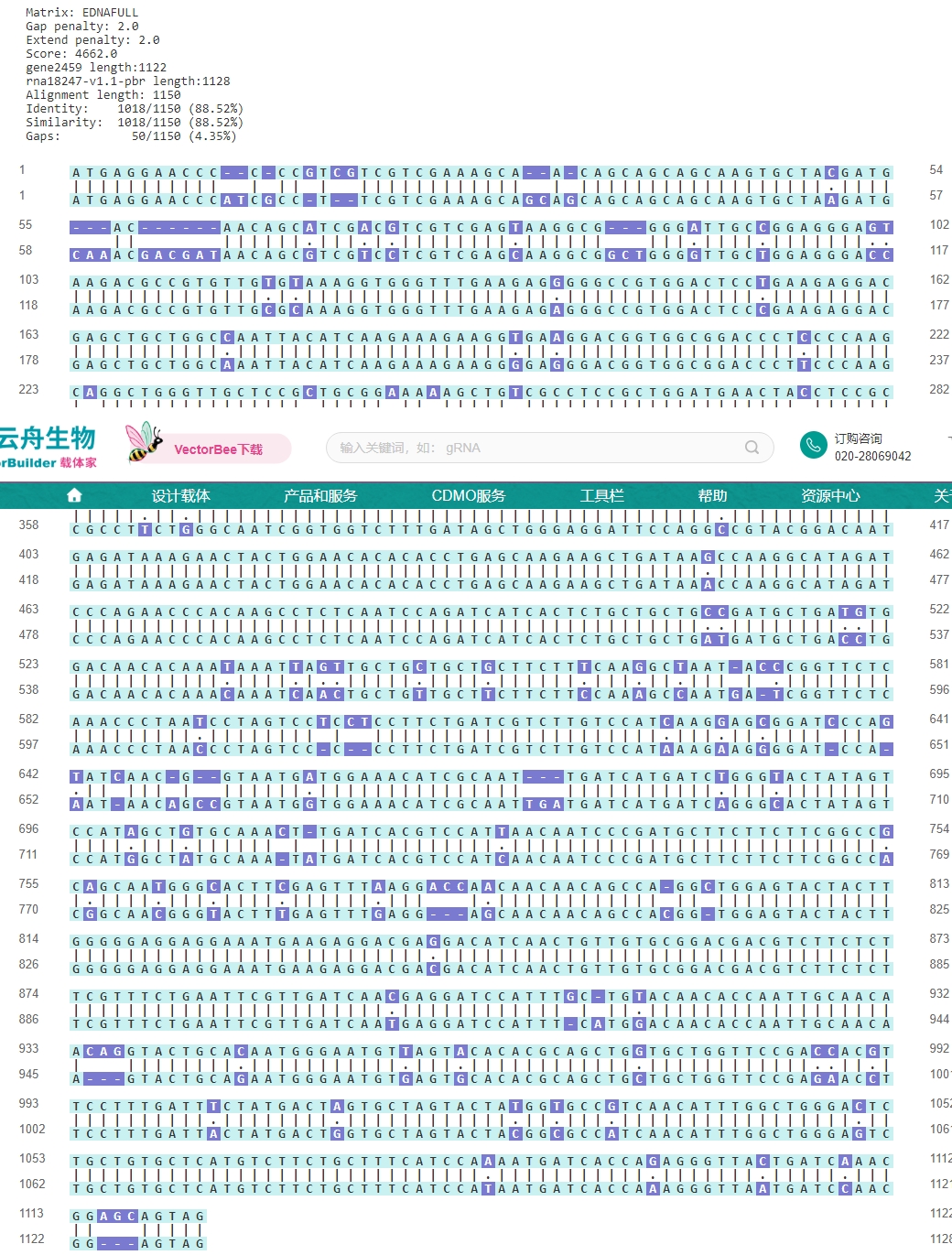


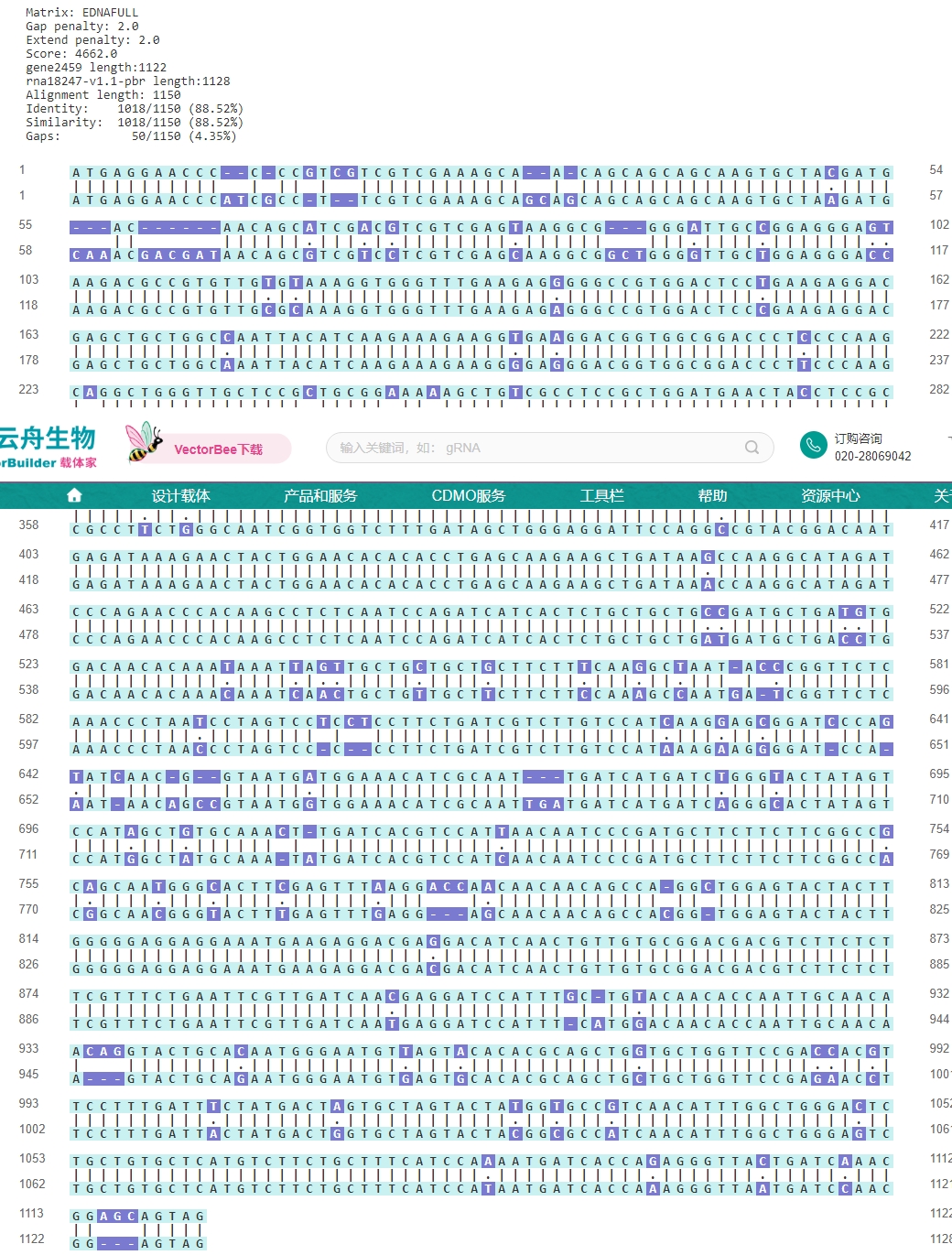


The results of the comparison of gene2459 with the CDS region of the sequence of rna18247-v1.1-pbr showed a sequence similarity of 88.52%.


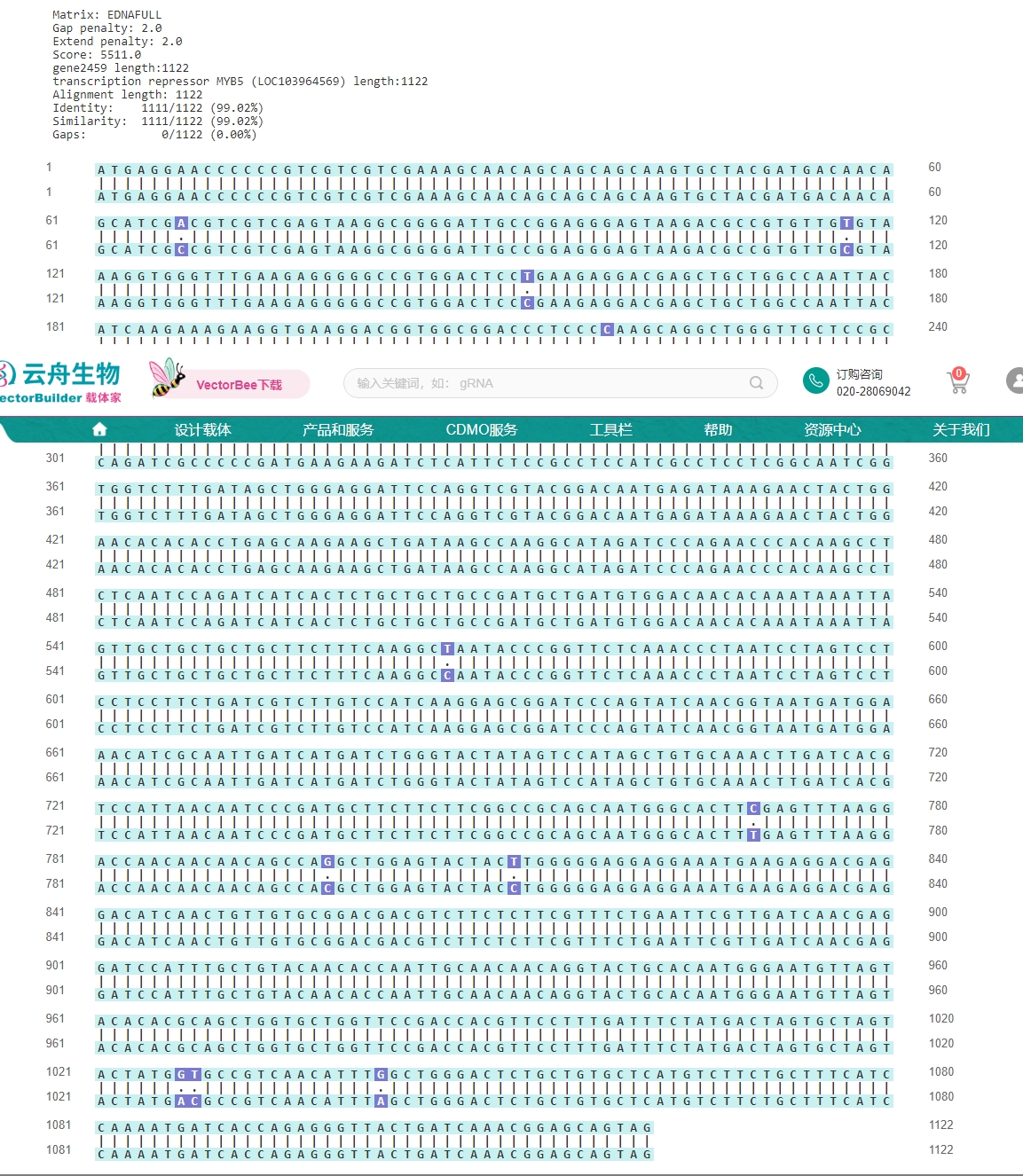

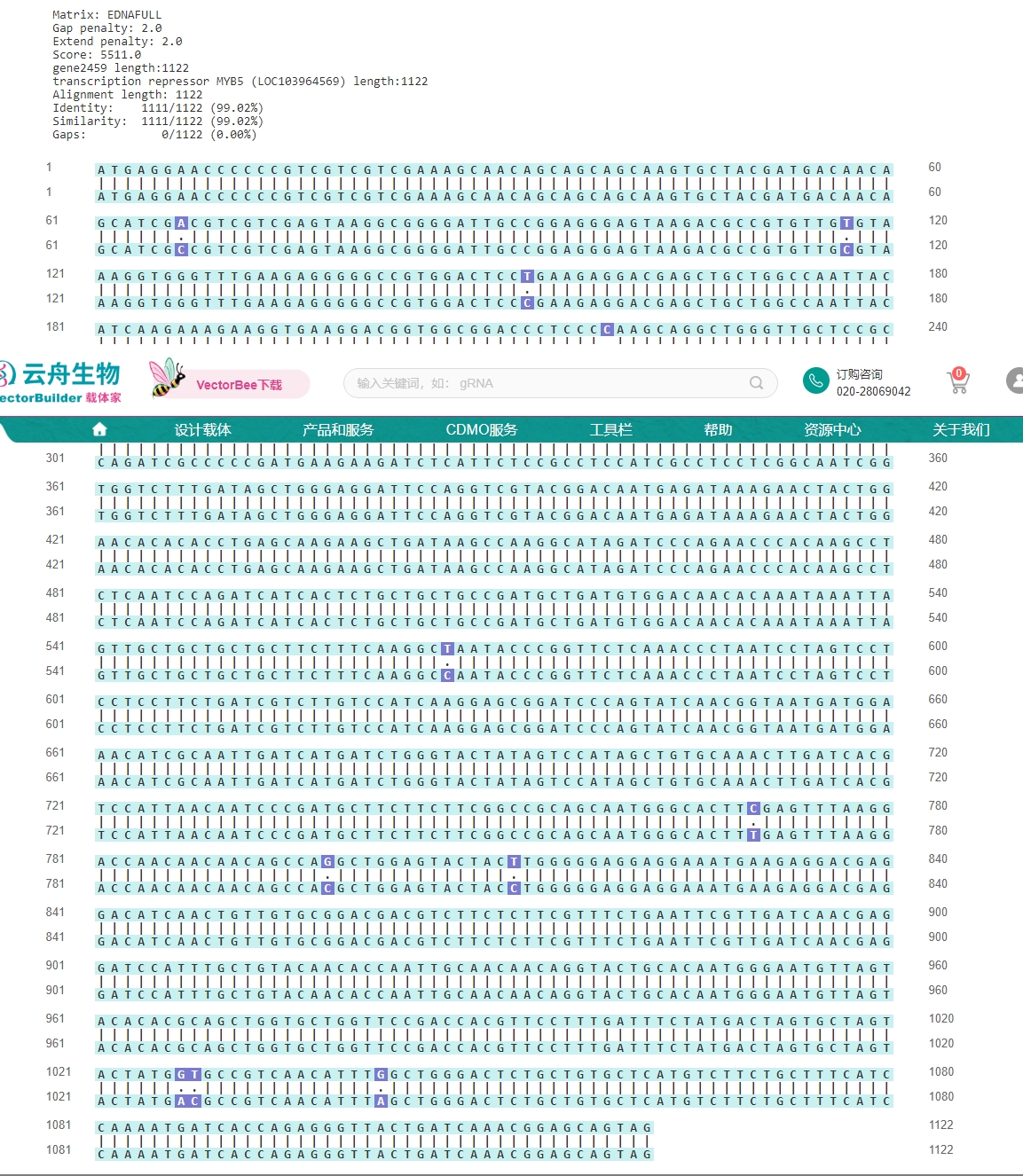


The results of the comparison of gene2459 with the CDS region of the sequence of Pyrus x bretschneideri transcription repressor MYB5 (LOC103964569) showed a sequence similarity of 99.02%.


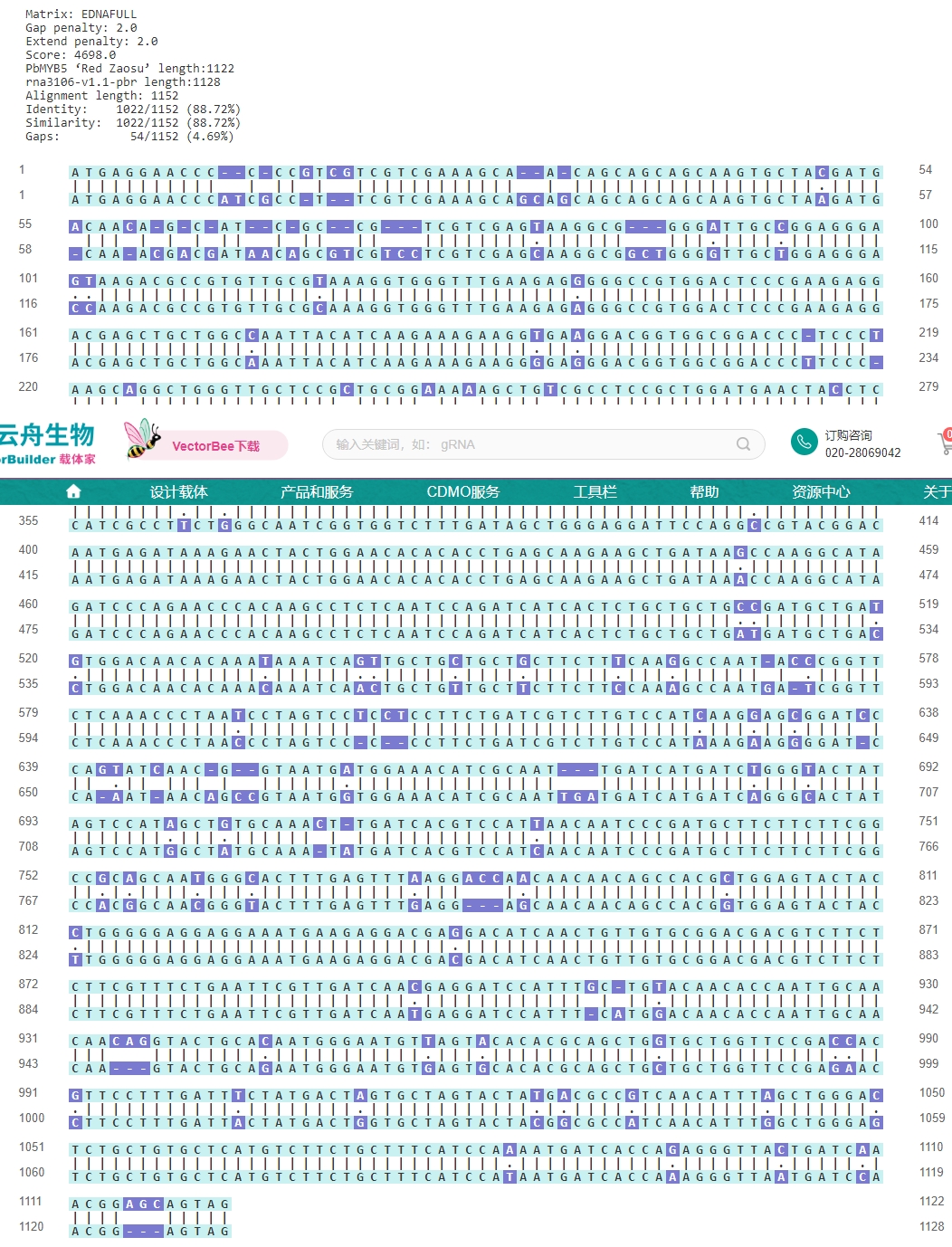


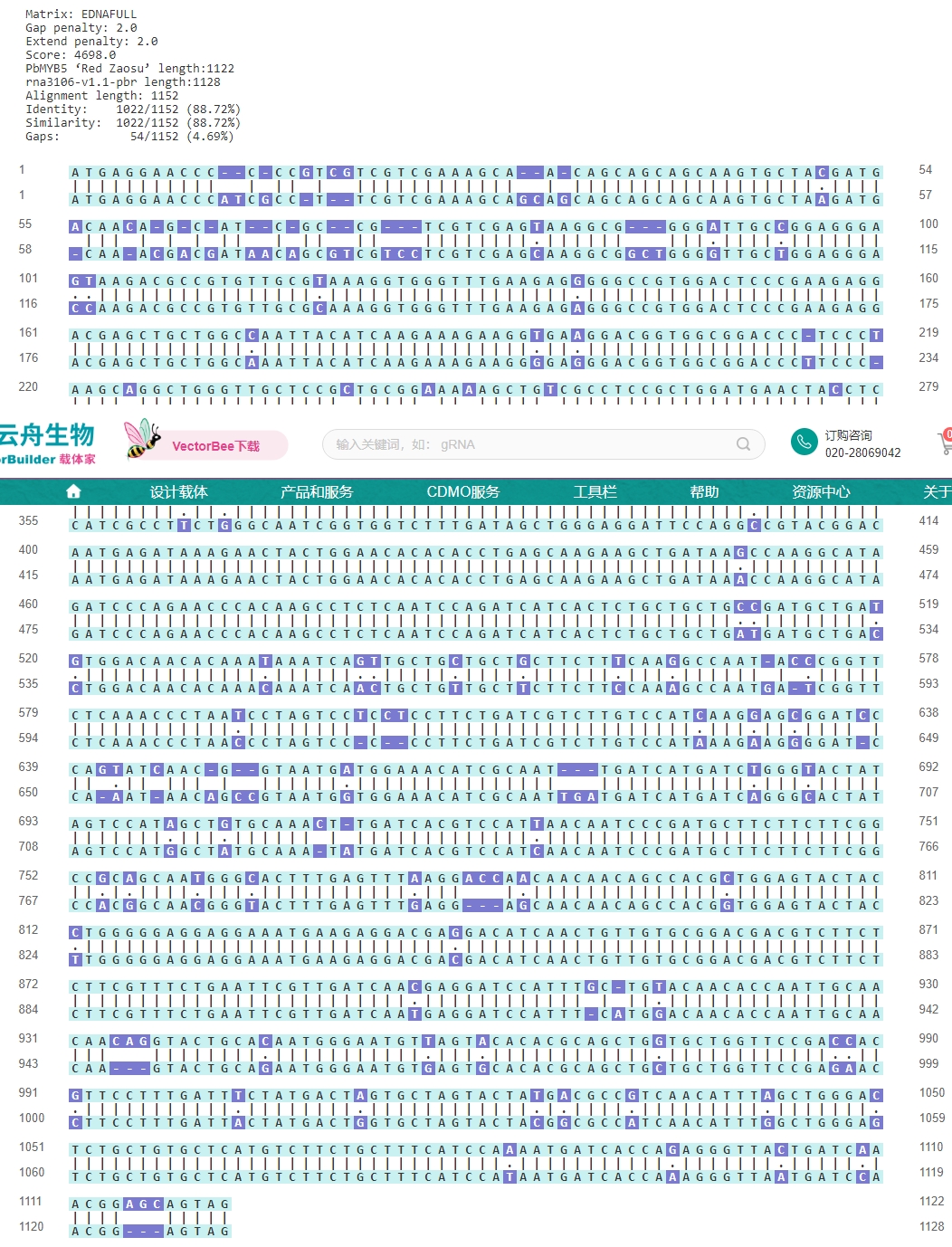


Alignment of PbMYB5 cloned from ‘Red Zaosu’ pear pericarp with the CDS region of the sequence of rna3106-v1.1-pbr showed a sequence similarity of 88.72%.


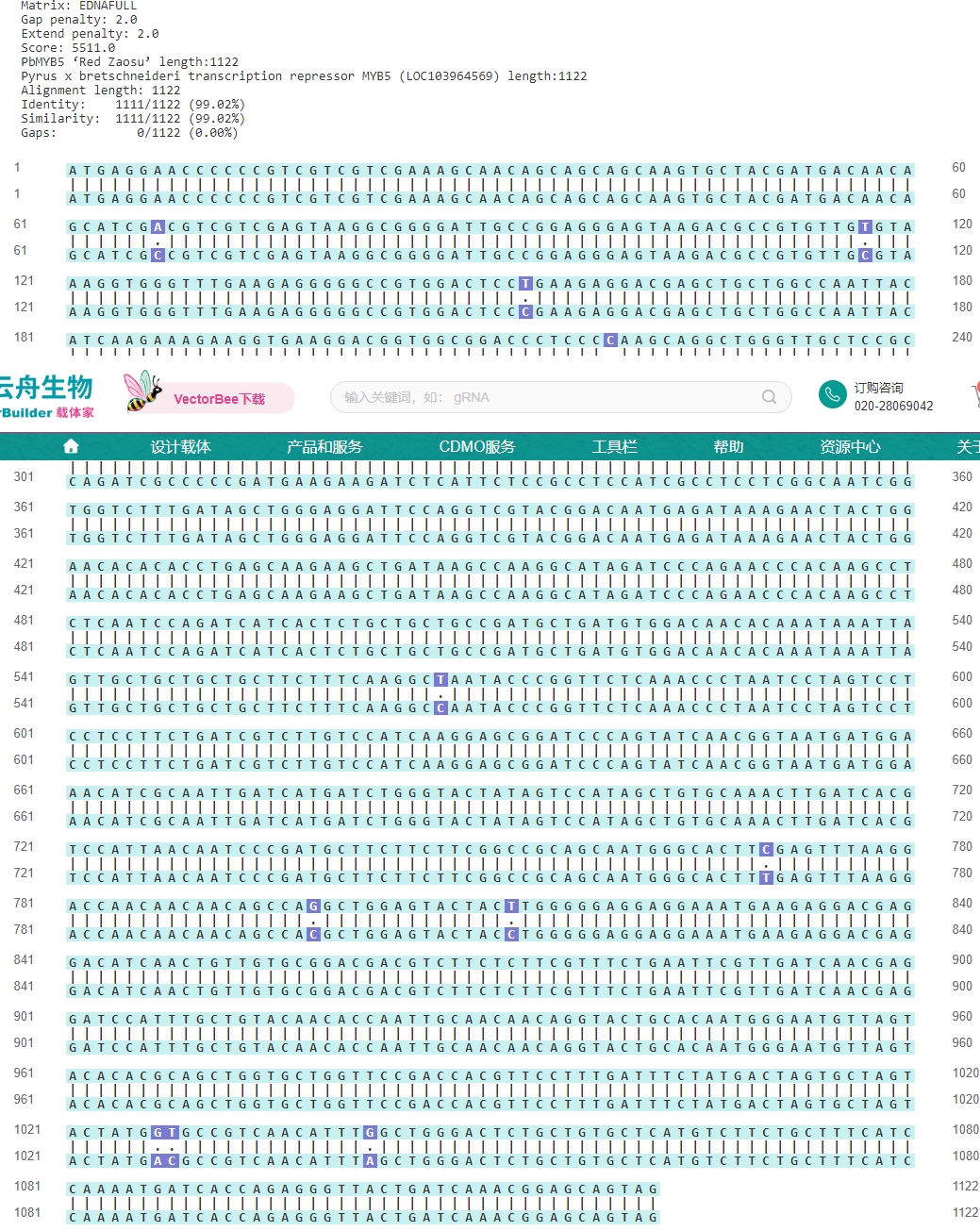

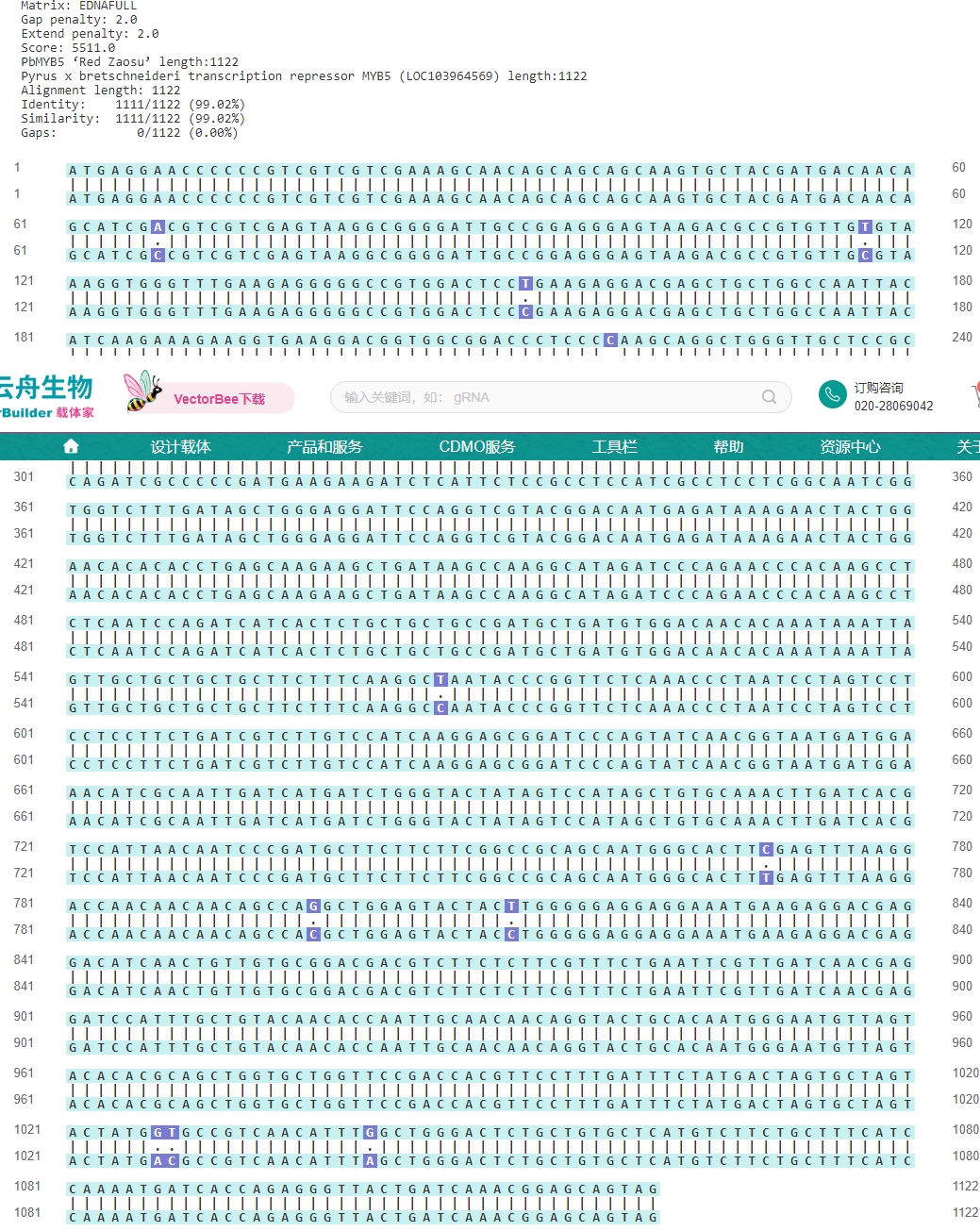


Alignment of PbMYB5 cloned from ‘Red Zaosu’ pear pericarp with the CDS region of the sequence of Pyrus x bretschneideri transcription repressor MYB5 (LOC103964569) showed a sequence similarity of 99.02%.

4. In summary, it can be seen that the sequence of gene2459 is highly consistent with the CDS sequence of Pyrus x bretschneideri transcription repressor MYB5 (LOC103964569) in the NCBI database, and primers were designed for the gene2459CDS region to ‘ Red Zaosu' pear pericarp cDNA as template and cloned to obtain PbMYB5.
